# Overexpression of RuBisCO form I and II genes in *Rhodopseudomonas palustris* TIE-1 augments polyhydroxyalkanoate production heterotrophically and autotrophically

**DOI:** 10.1128/aem.01438-24

**Published:** 2024-08-20

**Authors:** Tahina Onina Ranaivoarisoa, Wei Bai, Rengasamy Karthikeyan, Hope Steele, Miriam Silberman, Jennifer Olabode, Eric Conners, Brian Gallagher, Arpita Bose

**Affiliations:** 1 Department of Biology, Washington University in St. Louis, St. Louis, Missouri, USA; 2 LifeFoundry, San Jose, California, USA; Michigan State University, East Lansing, Michigan, USA

**Keywords:** *Rhodopseudomonas palustris *TIE-1, bioplastics, polyhydroxyalkanoate, *phaR*, *phaZ*, glycogen, *nifA*, RuBisCO

## Abstract

With the rising demand for sustainable renewable resources, microorganisms capable of producing bioproducts such as bioplastics are attractive. While many bioproduction systems are well-studied in model organisms, investigating non-model organisms is essential to expand the field and utilize metabolically versatile strains. This investigation centers on *Rhodopseudomonas palustris* TIE-1, a purple non-sulfur bacterium capable of producing bioplastics. To increase bioplastic production, genes encoding the putative regulatory protein PhaR and the depolymerase PhaZ of the polyhydroxyalkanoate (PHA) biosynthesis pathway were deleted. Genes associated with pathways that might compete with PHA production, specifically those linked to glycogen production and nitrogen fixation, were deleted. Additionally, RuBisCO form I and II genes were integrated into TIE-1’s genome by a phage integration system, developed in this study. Our results show that deletion of *phaR* increases PHA production when TIE-1 is grown photoheterotrophically with butyrate and ammonium chloride (NH_4_Cl). Mutants unable to produce glycogen or fix nitrogen show increased PHA production under photoautotrophic growth with hydrogen and NH_4_Cl. The most significant increase in PHA production was observed when RuBisCO form I and form I & II genes were overexpressed, five times under photoheterotrophy with butyrate, two times with hydrogen and NH_4_Cl, and two times under photoelectrotrophic growth with N_2_ . In summary, inserting copies of RuBisCO genes into the TIE-1 genome is a more effective strategy than deleting competing pathways to increase PHA production in TIE-1. The successful use of the phage integration system opens numerous opportunities for synthetic biology in TIE-1.

**IMPORTANCE:**

Our planet has been burdened by pollution resulting from the extensive use of petroleum-derived plastics for the last few decades. Since the discovery of biodegradable plastic alternatives, concerted efforts have been made to enhance their bioproduction. The versatile microorganism *Rhodopseudomonas palustris* TIE-1 (TIE-1) stands out as a promising candidate for bioplastic synthesis, owing to its ability to use multiple electron sources, fix the greenhouse gas CO_2_, and use light as an energy source. Two categories of strains were meticulously designed from the TIE-1 wild-type to augment the production of polyhydroxyalkanoate (PHA), one such bioplastic produced. The first group includes mutants carrying a deletion of the *phaR* or *phaZ* genes in the PHA pathway, and those lacking potential competitive carbon and energy sinks to the PHA pathway (namely, glycogen biosynthesis and nitrogen fixation). The second group comprises TIE-1 strains that overexpress RuBisCO form I or form I & II genes inserted via a phage integration system. By studying numerous metabolic mutants and overexpression strains, we conclude that genetic modifications in the environmental microbe TIE-1 can improve PHA production. When combined with other approaches (such as reactor design, use of microbial consortia, and different feedstocks), genetic and metabolic manipulations of purple nonsulfur bacteria like TIE-1 are essential for replacing petroleum-derived plastics with biodegradable plastics like PHA.

## INTRODUCTION

Recent improvements in genetic engineering tools have enabled scientists to systematically engineer organisms that produce various value-added chemicals, including biofuels, therapeutic products, food, and bioplastics (1–3). At first, most of these engineering efforts were focused on widely used model organisms, such as *Escherichia coli*, *Saccharomyces cerevisiae,* and *Synechococcus* sp. MIT9509 (4–8). This emphasis resulted in an array of genetic tools that have been effectively developed for valuable biomolecule biosynthesis and for conducting physiological studies (8–22). However, the reliance on organic carbon as the primary carbon source poses a limitation for heterotrophic model organisms, contributing to elevated bioproduction costs (15–17). Recent studies have highlighted numerous advantages in utilizing non-model organisms for bioproduction (23–25). Over the past decade, one such group of microbes that has gained attention is the purple nonsulfur bacteria exemplified by *Rhodopseudomonas palustris* TIE-1 (TIE-1) (26–28), *Rhodospirillum rubrum* (29, 30) and *Rhodomicrobium* (21, 31). TIE-1, a Gram-negative purple nonsulfur photosynthetic bacterium is renowned for its versatile metabolism, rendering it an excellent host for diverse bioproduction and pathway studies (26, 27). TIE-1 exhibits four primary metabolisms: chemoautotrophy, photoautotrophy, chemoheterotrophy, and photoheterotrophy (26, 27). These different metabolisms enable TIE-1 to use a wide variety of carbon sources such as carbon dioxide (CO_2_) and many organic acids. TIE-1 can use ammonium salts such as ammonium chloride (NH_4_Cl) or fix nitrogen from dinitrogen gas (N_2_) as a nitrogen source (26). Moreover, it can use multiple electron sources including hydrogen (H_2_) or ferrous iron (Fe (II)). One of the most appealing features of TIE-1 is its ability to acquire electrons directly from a poised electrode, which enables us to use it in microbial electrosynthesis (MES) (27, 32–35). MES is a system in which microorganisms are used to produce valuable compounds using their ability to obtain electrons from the bioelectrical reactor (33, 35, 36). Using electrons from a poised electrode or a solar panel-powered MES system, TIE-1 produces biodegradable plastic and biofuel using CO_2_ as a carbon source, N_2_ as a nitrogen source, and light as an energy source (27, 37). These represented the first steps toward a sustainable and carbon-neutral process for bioproduction using TIE-1 in MES. Besides its ability to utilize various substrates, TIE-1’s metabolic diversity also makes it an extraordinary model organism for pathway investigation (38, 39). For example, the use of RuBisCO mutants allowed us to study the association between the Calvin–Benson–Bassham (CBB) cycle in carbon fixation and extracellular electron transport (40). Similarly, a TIE-1 ∆*pioABC* mutant was used to investigate the electron uptake mechanism during photoferrotrophic and electrotrophic growth conditions (38). During these studies, mutants were first generated and grown under heterotrophic growth conditions in which the electron uptake machineries were not involved. These strains were then switched to autotrophic and electron uptake growth conditions to further understand their role in metabolism (38, 40). Not only do these studies provide insights into TIE-1’s metabolism, but they also open doors for a deeper understanding of other closely related purple nonsulfur bacteria, such as *Rhodopseudomonas palustris* CGA009, which has been studied extensively for biohydrogen production (41, 42).

The available genetic tools for TIE-1 are limited compared to widely used model organisms, with most tools being based on homologous recombination (34, 40). Fig. S1 illustrates the two-step homologous recombination process for achieving gene integration in TIE-1. Although this process results in markerless strains, it is time-consuming, with an efficiency lower than 50% (34). To reduce processing time and enhance efficiency, we explored phage recombination techniques for gene integration. This technique has gained attention in various bacteria, including *Methanosarcina* (43) and *Mycobacterium smegmatis* (44), due to its simple design and high efficiency (45, 46). The φC31 phage recombinase is commonly utilized due to its ability to function independently of a helper protein, and the recombination is unidirectional (47). For example, in *Clostridium ljungdahlii*, a whole butyric acid synthesis pathway was integrated into its genome by φC31 recombinase (45). In *Methanosarcina* spp., the φC31 recombinase achieved genome editing efficiency that is 30 times higher than that of homologous recombination (43) .

Among many value-added chemical products obtained from advanced genome engineering tools, bioplastics from microorganisms have become an attractive product (reviewed in (48)). This is particularly pertinent due to the detrimental environmental impact of excessive plastic usage in recent years. Bioplastics preserve the advantageous properties of petroleum-based plastics, such as high durability, moldability, water, and heat resistance, while also offering biocompatibility, emerge as a promising alternative (49–51). Moreover, numerous microorganisms possess the natural ability to degrade bioplastics, including the polyhydroxyalkanoate (PHA) family, either aerobically or anerobically, typically within a span of 5 to 6 weeks (50–52). However, the high feedstock cost remains an obstacle to the bioplastic’s competitiveness in the market (reviewed in (53, 54)). This issue can be addressed by using photoautotrophic microbes that can use cheap alternatives and waste feedstock (such as CO_2_) (27).

PHA stands out as the most extensively studied bioplastic (48, 54). The PHA pathway consists mainly of *phaA, phaB, phaC,* and *phaZ* genes. *phaA* encodes a ß-keto thiolase, while *phaB* encodes an acetoacetyl-CoA reductase (27). PhaC catalyzes the polymerization of PHA, whereas PHA depolymerase, PhaZ, catalyzes its mobilization during carbon starvation (27). Additional proteins, such as PhaP and PhaR, have also been reported to contribute to the maturation and regulation of PHA granules (55, 56). In *Paracoccus denitrificans,* PhaR is characterized as a repressor of PHA synthesis and acts by binding to the intergenic region of the *phaC-phaP* and *phaP-phaR* genes (55). However, PhaR is proposed to be an activator for PHA synthesis in *Cupriavidus necator* in a PhaP-dependent and -independent manner. Deletion of the *phaR* gene decreased PHA production in this organism (56). We have previously reported that TIE-1 possesses one *phaR* gene (Rpal_0531) using bioinformatics (27).

To improve PHA production, several gene manipulations have been undertaken directly on PHA pathway genes or genes from other pathways that may impact or compete with the PHA biosynthesis pathway (57–59). For instance, a study revealed that the deletion of the *phaZ* gene in *Sinorhizobium meliloti* increased PHA production compared to wild-type when utilizing formate generated through electrochemical CO_2_ reduction (57). This suggests that prevention of PHA degradation results in intracellular accumulation of PHA. Metabolic pathways such as glycogen production and nitrogen fixation are also potential competitors for bacterial PHA production (58). In *Synechocystis* sp. PCC 6803, PHA accumulation is reported to be linked to glycogen production under prolonged nitrogen starvation conditions. Mutants lacking the glycogen phosphorylase genes showed impaired PHA accumulation, supporting the link between glycogen and PHA synthesis under nitrogen starvation (NaNO_3_) conditions (58). Nitrogen fixation, which is a pathway highly demanding of electrons, could also present another competition to PHA production (60). Furthermore, PHA accumulation has also been reported to be induced by nitrogen deprivation ((N_2_) or NH_4_Cl) in many bacteria (41, 61, 62).

In another study, increase in PHA production was achieved by enhancing carbon fixation through the Calvin–Benson–Bassham (CBB) cycle in *Ralstonia eutropha* (now *C. necator*) (60). This enhancement involved the heterologous overexpression of the RuBisCO gene from *Synechococcus sp.* PCC 7002 in *Ralstonia eutropha*. Consequently, the overexpression resulted in a substantial increase in cell density, measured by optical density, by up to 89.2%. Additionally, there was a significant augmentation of the mass percent of PHA production, reaching up to 99.7% (60).

In this study, we used the φC31 integration system to integrate additional copies of the RuBisCO form I and II genes driven by the *P_aphII_* constitutive promoter into the TIE-1 genome. Additionally, to increase PHA accumulation in TIE-1, we created mutants that lack key genes in the PHA pathway, particularly focusing on the *phaZ* and the *phaR* genes. The behavior of the mutants lacking the *phaR* helped us elucidate whether *phaR* is an activator or repressor of the PHA pathway in TIE-1. In addition to exploring genes within the PHA pathway, we also examined the impact of deleting genes in pathways that might potentially compete with the PHA synthesis pathway. These include the mutants that have been previously studied in biobutanol production in the TIE-1: Δ*gly* mutant lacking glycogen synthase and the mutant lacking the two NifA regulators *nifA*, Rpal_1624 & Rpal_5113, of the nitrogen fixation pathway of TIE-1 (37). We also hypothesize that like *R. eutropha,* overexpressing RuBisCO genes (form I and II) in TIE-1 could increase intracellular carbon abundance and hence increase PHA accumulation. We tested PHA production in all six strains and wild-type under a variety of growth conditions, including non-nitrogen-fixing conditions with NH_4_Cl (referred to as non-nitrogen-fixing conditions throughout) and nitrogen-fixing conditions with N_2_ gas (referred to as nitrogen-fixing conditions throughout).

Our results show that the deletion of the *phaR* gene increased PHA production (dry cell weight % wt/wt) when TIE-1 was grown photoheterotrophically with butyrate under non-nitrogen-fixing conditions. We also observed an increase in PHA production (dry cell weight % wt/wt) from the *Δgly* and *ΔnifA* under photoautotrophic growth conditions with H_2_ and under non-nitrogen-fixing conditions. PHA production increased in TIE-1 strains overexpressing RuBisCO form I and form I & II genes under photoheterotrophy with butyrate irrespective of the nitrogen source used and photoautotrophy with H_2_ under non-nitrogen-fixing conditions as well as photoelectrotrophically under nitrogen fixing conditions. We show that both gene deletion and overexpression can enhance PHA production by TIE-1. This work advances TIE-1 as a model organism for potential commercialization for PHA production and provides valuable insights for future genetic engineering endeavors aimed at enhancing bioplastic production in other purple nonsulfur bacteria.

## RESULTS

### Generating TIE-1 mutants and suicide plasmids with the different antibiotic markers

To improve polyhydroxyalkanoate (PHA) production, we constructed a mutant lacking the regulator of PHA pathway (Δ*phaR*) and a mutant lacking PHA depolymerase (Δ*phaZ*) (Fig. S1 and S2). The mutant lacking the glycogen synthesis gene (Δ*gly*) as well as the double mutant lacking the nitrogen-fixing regulator *nifA* genes (Δ*nifA*1 Δ*nifA*2; referred as *DnifA* throughout) were constructed previously (37) . These strains are listed in Table 1, and the plasmids used to construct them are listed in Table 2.

**TABLE 1:** Strains used in this study

Strain	Relative characteristics	Function	Source
AB415	Wild-type *Rhodopseudomonas palustris* TIE-1	Wild-type TIE-1	(26)
WB065	Wild-type TIE-1 with *attB*	For designing RuBisCO strains	This study
WB068	Wild-type TIE-1 with φ*C31* integrase; *P_lac_*; *lacIq*; *attB*	For designing RuBisCO strains	This study
WB090	Wild-type TIE-1 with *mCherry*; *P_aphII_* promoter; *fd* terminator; *attL*; *attR*	For designing RuBisCO strains	This study
WB091	Wild-type TIE-1 with *loxp-mCherry-loxp*; *P_aphII_* promoter; *fd* terminator; *attL*; *attR*	For designing RuBisCO strains	This study
AB188	TIE-1 Δ*gly* mutant	TIE-1 mutant lacking the glycogen synthase gene	(37), this study
AB187	TIE-1 Δ*nifA* double mutant	TIE-1 mutant lacking the *nifA*_1&2_ genes	(37), this study
AB186	Δ*phaR*	TIE-1 mutant lacking the *phaR* gene	This study
AB189	Δ*phaZ*	TIE-1 mutant lacking the *phaZ* gene	This study
AB199	Wild-type TIE-1 with RuBisCO form I gene overexpressed	Engineered TIE-1 for PHA overproduction	This study
AB200	Wild-type TIE-1 with RuBisCO form I and II genes overexpressed	Engineered TIE-1 for PHA overproduction	This study

**TABLE 2:** Plasmids used in this study

Plasmid	Relative characteristics	Source
pJQ200KS	ori *P15A*; Gen^r^	(63)
pAB314	pJQ200KS with *GlmUSX*_UP and *GlmUSX*_DN	(34)
pAB356	Cm^r^	(64)
pAB357	Kan^r^	(64)
pAB358	Tc^r^	(64)
pAB359	Amp^r^	(64)
pSRKGm	*P_lac_*; *lacIq*; ori *pBBR1*; Gen^r^	(64)
pWB083	pAB314 with *attB*	This study
pWB081	pJQ200KS with *mCherry*; *P_aphII_* promoter; *fd* terminator and *attP*	This study
pWB084	pSRKGm with φ*C31* integrase	This study
pWB086	pAB314 with φ*C31* integrase; *P_lac_*; *lacIq*; *attB*	This study
pWB088	pJQ200KS with φ*C31* integrase; *P_aphII_* promoter; *fd* terminator and *attP*	This study
pWB091	pJQ200KS with Kan^r^	This study
pWB092	pJQ200KS with Cm^r^	This study
pWB107	RuBisCO form I and form II gene overexpression	This study
pWB108	RuBisCO form I gene overexpression	This study
pAB863	pJQ200KS with *phaR* 1 kb up and 1 kb down for deletion of *phaR*	This study
pAB868	pJQ200KS with *phaZ* 1 kb up and 1 kb down for deletion of *phaZ*	This study

All the mutants were constructed by using a suicide plasmid (*pJQ200KS*) carrying a gentamicin resistance cassette. Using gentamycin has two disadvantages: 1) wild-type TIE-1 has a remarkably high resistance to gentamicin with a minimum inhibitory concentration (MIC) of 200 µg/mL and 2) gentamicin is expensive (65). For enhanced competitiveness in large-scale production of PHA, opting for other antibiotics with lower MIC values than gentamicin could offer a more cost-effective alternative. Accordingly, we constructed suicide plasmids containing two widely used antibiotic resistance markers: chloramphenicol (MIC of 100 µg/mL) or kanamycin (MIC of 50 µg/mL), using *pJQ200KS* as the backbone, as listed in Table 2 and shown in Fig. S3.

### Engineering TIE-1 using a phage integration system to increase PHA production in TIE-1

**Fig 1:**
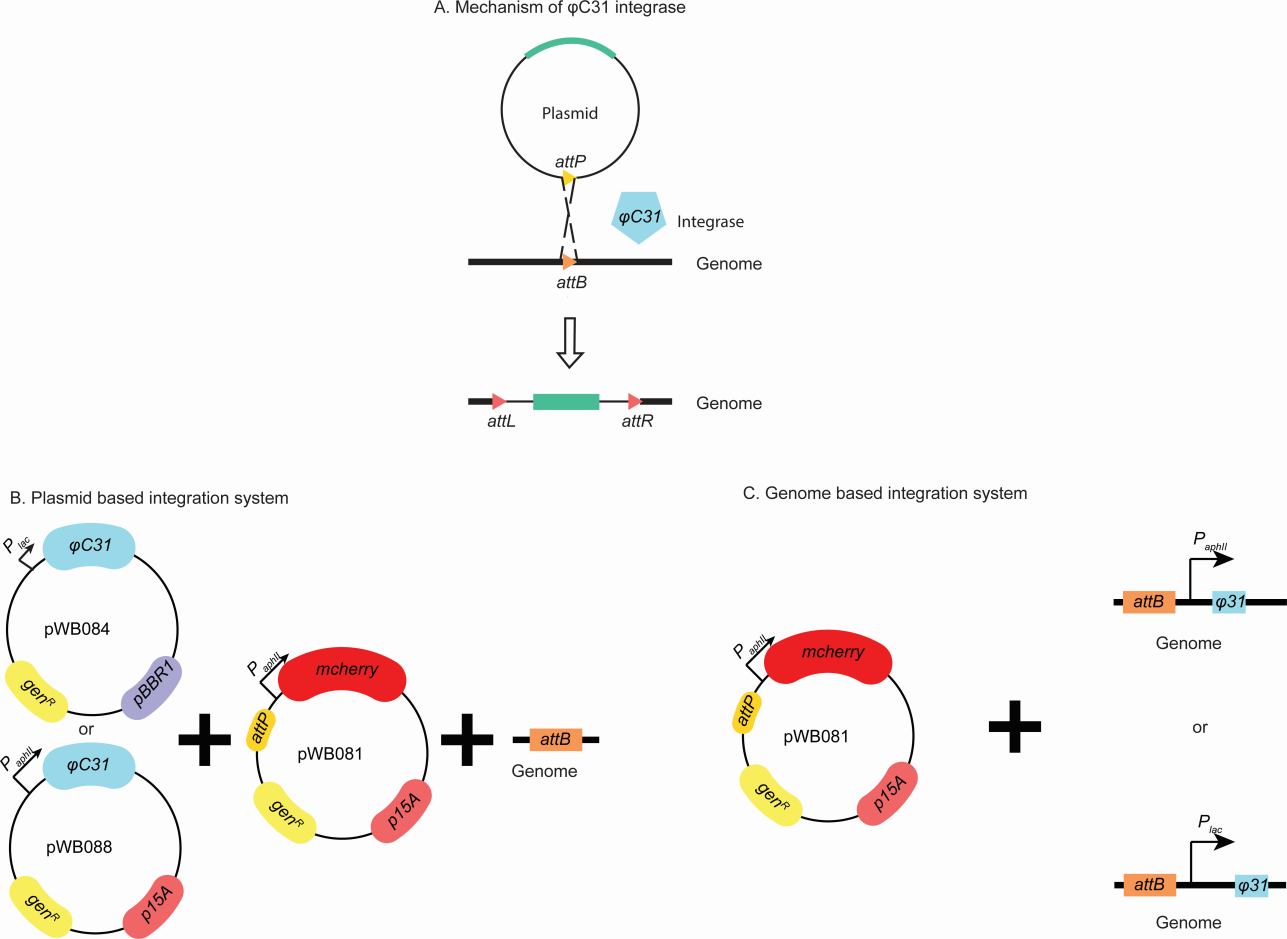
Phage integration system. (A) φC31 integrase mechanism; (B) plasmid-based integration system; (C) genome-based integration system. *P_lac_, P_lac_* promoter; *_PaphII_, P_aphII_* promoter; *mCherry*, red fluorescent protein, *gen^R^*, gentamicin resistance; *p15A*, the origin of replication; *pBBR1*, broad host origin of replication; *attP, attP* site for φC31integrase; *attB, attB* site for φC31 integrase; φC31, φC31 integrase.

As previously discussed, homologous recombination presents a time-intensive process and remains so far the only successful genetic tool for TIE-1. To address these challenges, we devised a phage integration system for incorporation of genes into its genome. Fig. 1 delineates the essential components of the φC31 recombinase system: the *attB* site, *attP* site, and the φC31 integrase.

**Fig 2:**
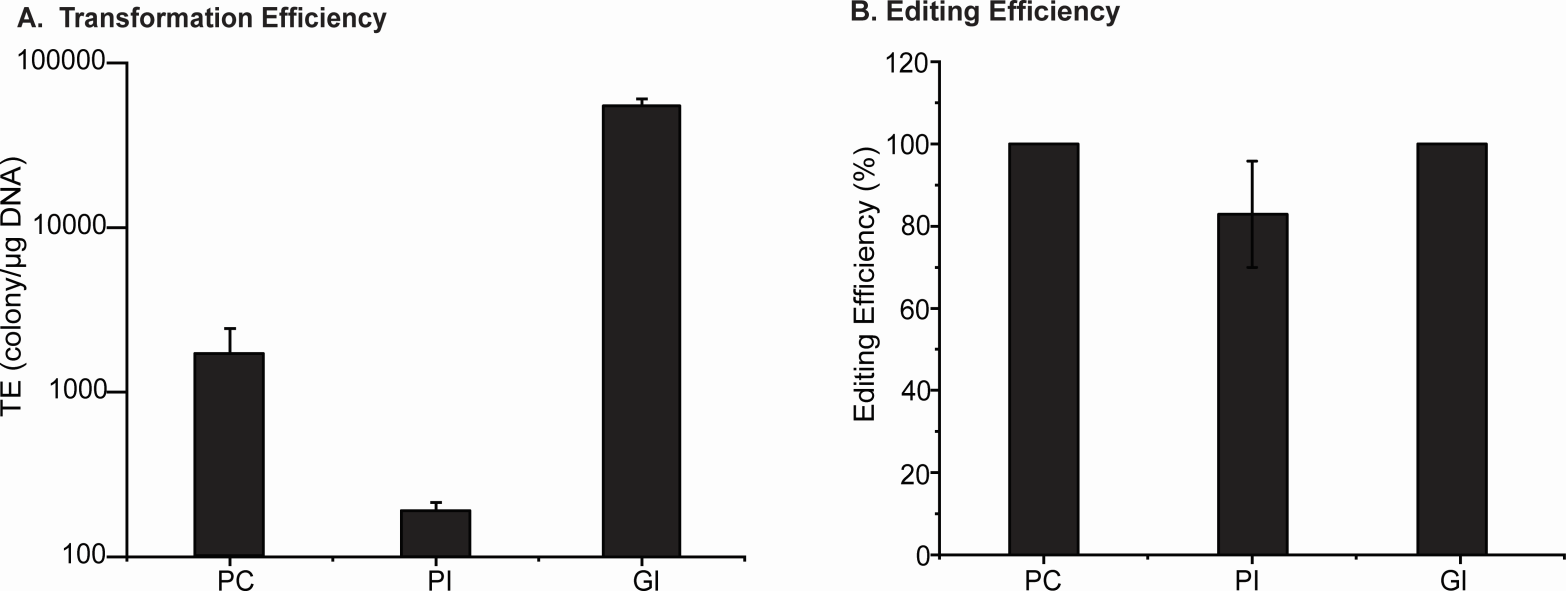
(A) Transformation efficiency normalized to the plasmid amount. (B) Editing efficiency. TE, transformation efficiency; PC, plasmid-based system with the constitutive promoter; PI, plasmid-based system with the inducible promoter; GI, genome-based system with the inducible promoter.

To compensate for the lack of an *attB* site in TIE-1, we inserted it into the genome, as described in Materials and Methods. The *attP* site was introduced into a suicide plasmid with a constitutively expressed *mCherry* gene under the *P_aphII_* promoter (pWB081). For the expression of φC31 integrase, the optimal way would be to use a temperature-sensitive plasmid (66). After the targeted genome editing, the removal of the φC31 integrase could be conveniently executed using this approach. Unfortunately, there is no known temperature-sensitive plasmid that replicates in TIE-1. Hence, we decided to build two different systems: (1) a plasmid-based system, where the integrase is introduced into TIE-1 by a plasmid (Fig. 1B), and (2) a genome-based system, where the integrase is integrated into the TIE-1 genome (Fig. 1C). The advantage of the plasmid-based system is its mobility, while the genome-based system is more stable and does not rely on antibiotics. Both an inducible promoter (*P_lac_*) and a strong constitutive promoter (*P_aphII_*) were tested. To summarize, as shown in Fig. 1, we have four different designs for the expression of the φC31 integrase: a) *P_aphII_*-driven φC31 integrase on a suicide plasmid (pWB088); b) *P_lac_*-driven φC31 integrase on a self-replicating plasmid (pWB084); c) *P_aphII_*-driven φC31 integrase on the TIE-1 genome (Fig. 1C(a)); and d) *P_lac_*-driven φC31 integrase on the TIE-1 genome (Fig. 1C(b)). After three separate trials, we were not able to obtain a genome-based system using the constitutive promoter (Fig. 1C(a)). Thus, we only present the results of the other three systems. The successful integration of *mCherry* was indicated by visualizing red fluorescence at an emission wavelength of 610 nm.

Upon confirming integration, we assessed the efficiency of the various systems by measuring the transformation efficiency (detailed calculation described in Materials and Methods) and the integration efficiency, which is defined as the percentage of colonies that have red fluorescence signals among all obtained colonies. As shown in Fig. 2A and B, the transformation efficiency normalized to the plasmid concentration is higher for the genome-based system (*P* < 0.001) (Fig. 1C). This higher efficiency could be due to the sufficiency of only one plasmid for the system to be functional. Between the two plasmid-based systems, the constitutively expressed φC31 reached higher efficiency (Fig. 1B, pWB088). This higher efficiency could be due to the independence from the use of the inducer, IPTG. For the integration frequency, the genome-based system (Fig. 1C) and the plasmid-based system both with the constitutive promoter (Fig. 1B, pWB088) and inducible promoter resulted in similar editing efficiencies between 80% and 100% (*P* = 0.13) (Fig. 2B). To summarize, the genome-based system with an inducible promoter results in highest transformation efficiency, while all three systems have similar editing efficiencies.

After successfully integrating *mCherry*, we wanted to use the phage integration tool to assist in improving PHA production in TIE-1. Previous research conducted on *Ralstonia eutropha* (now *Cupriavidus necator*) has shown that overexpression of RuBisCO resulted in improved PHA production (60). Thus, to improve PHA production, we integrated a *P_aphII_ -*driven RuBisCO form I alone or RuBisCO form I and form II together into the TIE-1 genome to obtain two new TIE-1 strains: *Ωrub(I*) and *Ωrub(I&II*). We were unable to obtain a plasmid with *P_aphII_ -*driven RuBisCO form II only. The successful integration of these genes and *P_aphII_* was analyzed by PCR amplification of the *P_aphII_* region and the RuBisCO gene form I and II, as indicated in Fig. S4. In addition, the whole genome of each constructed strain has been sequenced.

### Deletion of *phaR, phaZ*, or overexpression of the RuBisCO form I impaired the growth of TIE-1 during photoheterotrophic growth with butyrate

**Fig 3:**
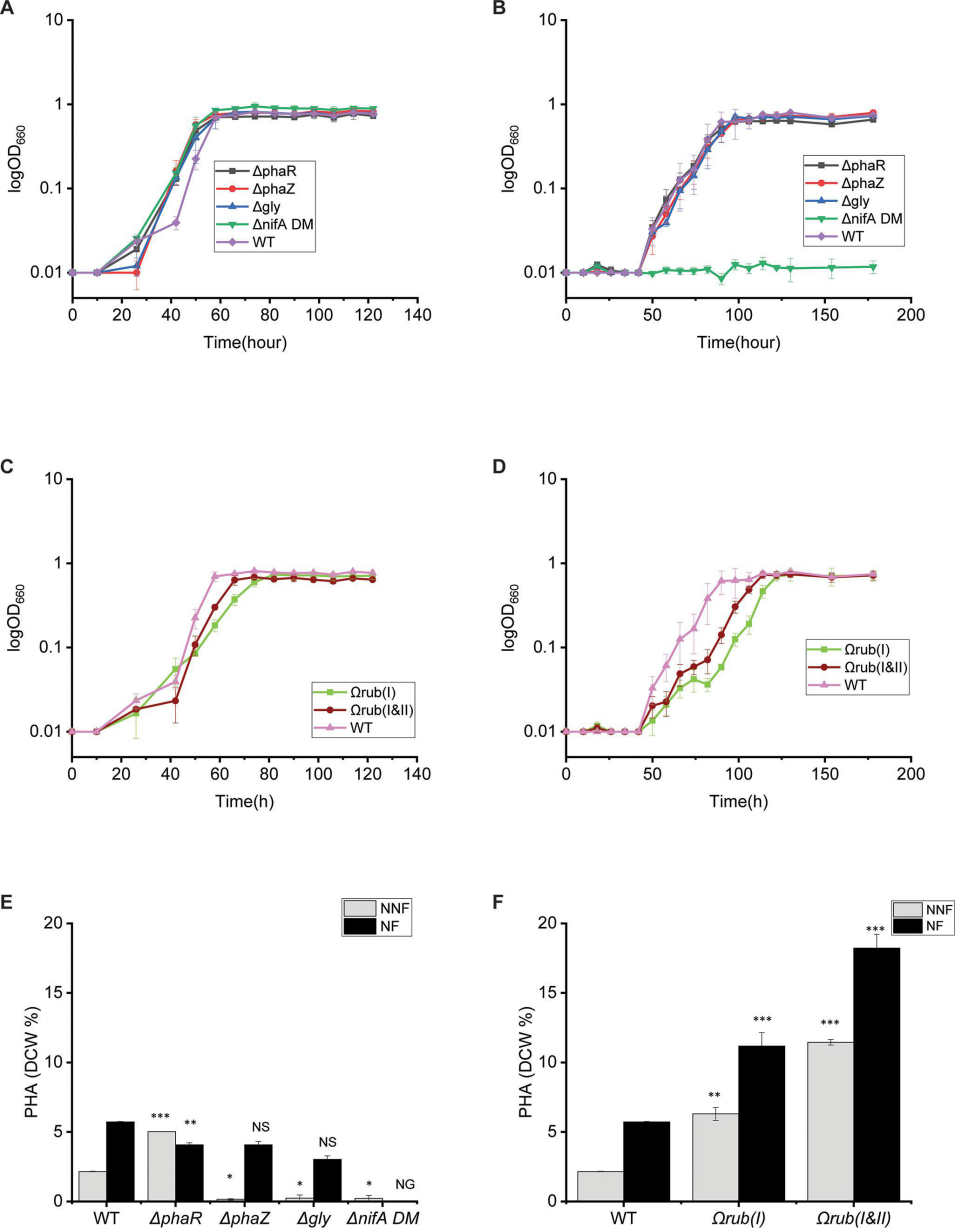
Growth and PHA production (dry cell weight % wt/wt) from different strains grown photoheterotrophically in fresh water basal media with butyrate. (A) Growth of all mutant strains under non-nitrogen-fixing conditions. (B) Growth of all mutant strains under nitrogen-fixing conditions (N_2_). (C) Growth of the RuBisCO engineered strains under non-nitrogen-fixing conditions. (D) Growth of all the RuBisCO engineered stains under nitrogen-fixing conditions. (E) PHA production from mutants and TIE-1 strains grown with butyrate under non-nitrogen-fixing and nitrogen-fixing conditions. (F) PHA production from the RuBisCO engineered and wild-type TIE-1 strains grown with butyrate under non-nitrogen-fixing and nitrogen-fixing conditions. NFF = non-nitrogen-fixing; NN = nitrogen-fixing. Error bars from growth curves represent the standard deviation calculated from three replicates, while error bars from PHA production depict the standard error derived from biological triplicates. The statistical differences in PHA production were calculated using two-tailed Student’s test against wild-type TIE-1. *P ≤ 0.05, **P < 0.01, and ***P < 0.001; NS, not significant; ND, not detected; NG, no growth.

Because TIE-1 has previously exhibited high PHA production under anoxic photoheterotrophic conditions with butyrate (27), we evaluated this growth condition using all the constructed strains. Under these anoxic growth conditions, light is used as an energy source and butyrate is used both as an electron and a carbon source. For all growth conditions explored, we determined the effect of nitrogen fixation by supplying N_2_ gas (nitrogen-fixing) or ammonium chloride (NH_4_Cl) salt (non-nitrogen-fixing). The growth parameters were obtained both from 1 mM (shown in Table 3; Fig. 3A and B) and 10 mM (shown in Table S1) butyrate. A 1 mM concentration was chosen to allow a clear growth trend without reaching saturation of the OD_660_. To collect enough biomass, PHA production was only obtained from growth with 10 mM butyrate. Our results show that the growth with 1 mM butyrate did not reveal a significant difference between the various constructed strains (Fig. 3A and B). Deleting *phaR* or *phaZ* increased the generation time of TIE-1 by around 3 hours compared to wild-type when grown photoheterotrophically with 10 mM butyrate under non-nitrogen-fixing conditions (Table S1) (*P* = 0.01). However, a slightly shorter generation time (around 3 hours shorter than that of wild-type) was obtained from the Δ*gly* and the Δ*nifA* under these conditions (Table S1) (*P* = 0.007). Under nitrogen-fixing conditions, the Δ*phaZ* mutant continued to show a growth defect observed as a longer generation time (3 hours longer than wild type), whereas the Δ*phaR* and Δ*gly* mutants showed the same generation time as that of wild-type (Table S1). As expected, Δ*nifA* was not able to grow under nitrogen-fixing conditions (Table 3; Table S1; Fig. 3B). We compared the growth of the engineered RuBisCO strains Ω*rub(I*) and Ω*rub(I&II*) under the same photoheterotrophic growth condition with butyrate. The engineered strain carrying only the overexpressed RuBisCO gene form I (Ω*rub(I*)) has a growth defect under both nitrogen-fixing and non-nitrogen-fixing conditions with 10 mM butyrate (Table S1), as well as with 1 mM butyrate (Table 3). This defect seems to be more noticeable under nitrogen-fixing growth conditions. The Ω*rub(I*) strains have an ~2X (*P* = 0.01) longer lag time than wild-type strains when grown photoheterotrophically with 10 mM butyrate under non-nitrogen-fixing conditions, and ~5X (*P* = 0.001) longer generation time under nitrogen-fixing conditions (Table S1). Lag time was also about 4 hours longer (*P* = 0.001) when the Ω*rub(I*) was grown with only 1 mM butyrate both under nitrogen and non-nitrogen-fixing conditions (Table 3).

**TABLE 3:** Growth parameter values of the knockout and engineered TIE-1 strains grown under different growth conditions^*a*^

	Δ*phaR*	*P*	Δ*phaZ*	*P*	Δ*gly*	*P*	Δ*nifA*	*P*	Ω*rub(I*)	*P*	Ω*rub(I&II*)	*P*	WT
Generation time G (h)													
But (NH_4_Cl)	6.58 (0.34)	0.39	5.45 (0.42)	**0.00**	6.81 (0.18)	0.96	6.58 (0.37)	0.40	6.79 (0.13)	0.92	6.49 (0.36)	0.25	6.80 (0.20)
But (N_2_)	10.18 (0.52)	**0.002**	9.57 (0.34)	**0.002**	8.03 (1.42)	0.99	NG	NG	8.48 (0.84)	0.42	7.0 (0.79)	0.094	8.04 (0.22)
H_2_ (NH_4_Cl)	14.98 (1.32)	**0.01**	13.20 (0.54)	**0.00**	9.74 (0.4)	0.442	11.16 (0.71)	0.154	9.7 (0.3)	0.37	10.51 (4.25)	0.89	10.14 (0.71)
H_2_ (N_2_)	88.80 (27.78)	0.52	64.90 (9.2)	0.36	75.29 (12.74)	0.971	NG	NG	149.9 (67.03)	0.13	99.77 (36.01)	0.35	75.74 (15.84)
Fe(II) (NH_4_Cl)	595 (30.31)	0.65	4620 (2000.5)	0.03	280.71 (57.66)	**0.021**	369.35 (115)	0.076	740.67 (265)	0.61	644.36 (119)	0.98	641.67 (160)
Fe(II) (N_2_)	ND	ND	NG	NG	235.14 (55.63)	0.498	NG	NG	371.49 (11.7)	**0.004**	205.33 (155)	0.57	262.1 (29.13)
PE (NH_4_Cl)	517.3 (48.1)	0.21	1212.75 (173.2)	0.0048	1008 (0)	0.016	822.9 (86.6)	0.0001	605.02 (3.49)	0.18	337.41 (10.7)	0.40	262.32 (9.11)
PE (N_2_)	646.8 (0)	0.71	3482.73 (3211)	0.28	42446.2 (84892)	0.37	NG	NG	1060.8 (105)	0.54	4207 (3316)	0.27	800.25 (115)
Maximum OD_660_													
But (NH_4_Cl)	0.78 (0.07)	0.35	0.86 (0.04)	0.22	0.81 (0.06)	0.79	0.89 (0.01)	**0.00**	0.71 (0.06)	**0.03**	0.69 (0.03)	**0.00**	0.82 (0.01)
But (N_2_)	0.653 (0.04)	**0.036**	0.67 (0.04)	0.113	0.70 (0.02)	0.107	NG	NG	0.78 (0.05)	0.32	0.80 (0.07)	0.28	0.74 (0.03)
H_2_ (NH_4_Cl)	2.48 (0)	**0.001**	2.495 (0.01)	**0.001**	1.36 (0.28)	0.454	1.64 (0.24)	0.12	1.98 (0.742)	0.26	2.22 (0.403)	0.07	1.17 (0.071)
H_2_ (N_2_)	1.73 (0.19)	0.642	1.963 (0.19)	0.11	1.89 (0.42)	0.409	NG	NG	1.73 (0.239)	0.68	1.3 (0.482)	0.31	1.65 (0.19)
Fe(II) (NH_4_Cl)	0.04 (0)	**0.002**	0.021 (0)	**0.016**	0.14 (0.008)	**0.00**	0.082 (0)	**0.00**	0.11 (0.012)	**0.00**	0.03 (0.001)	**0.01**	0.03 (0.0017)
Fe(II) (N_2_)	0.02 (0.01)	**0.013**	NG	**NG**	0.10 (0.02)	0.977	NG	NG	0.06 (0.002)	0.09	0.11 (0.029)	0.85	0.09 (0.012)
PE (NH_4_Cl)	0.27 (0.00)	0.332	0.160 (0.00)	**0.029**	0.17 (0)	**0.036**	0.17 (0.00)	**0.035**	0.26 (0.006)	0.29	0.29 (0.001)	0.34	0.38 (0.003)
PE (N_2_)	0.39 (0.005)	0.218	0.19 (0.00)	0.944	0.098 (0.002)	**0.01**	NG	NG	0.16 (0.002)	0.46	0.122 (0)	**0.04**	0.19 (0.001)
Time to reach maximum OD (h)													
But (NH_4_Cl)	98 (0.00)	**0**	114 (0.00)	**0**	74 (0.00)	**0**	74 (0.00)	**0**	82 (0.00)	**0**	74 (0.00)	**0**	74 (0.00)
But (N_2_)	122 (0)	**0.0065**	122.33 (0)	**0.000**	114 (0)	**0**	NG	NG	130 (0)	**0**	114 (0)	**0**	98 (0)
H_2_ (NH_4_Cl)	90.5 (0.71)	**0.00**	290.5 (0.70)	0.00	338.5 (0.71)	1	338.5 (0.71)	1	243 (135.76)	0.42	338.5 (0.71)	1	338.5 (0.71)
H_2_ (N_2_)	472.67 (0.58)	1	472.7 (0.57)	1	472.67 (0.58)	1	NG	NG	472.67 (0.58)	1	472.67 (0.58)	1	472.67 (0.58)
Fe(II) (NH_4_Cl)	1680.33 (0.5)	**0.00**	1680.2 (0.29)	**0.00**	1320.33 (0.58)	**0.00**	1320.67 (1.1)	**0**	1319.67 (0.5)	**0.00**	936.33 (0.58)	0.23	935.67 (0.58)
Fe(II) (N_2_)	1704.33 (0.5)	**0.00**	NG	NG	1200.33 (0.58)	1	NG	NG	671.67 (0.58)	**0.00**	1200.33 (0.5)	0.37	1200.33 (0.58)
PE (NH_4_Cl)	336 (0)	**0.00**	336 (0)	**0.00**	336 (0)	**0.00**	336 (0)	**0.00**	336 (0)	**0.00**	336 (0)	**0.00**	336 (0)
PE (N_2_)	336 (0)	**0.00**	336 (0)	**0.00**	336 (0)	**0.00**	NG	NG	336 (0)	**0.00**	336 (0)	**0.00**	336 (0)
Lag time (h)													
But (NH_4_Cl)	10.80 (0.23)	**0.00**	10.92 (0.71)	**0.00**	10.11 (1.10)	0.11	10.77 (0.28)	**0.00**	14.32 (2.72)	**0.00**	11.52 (2.00)	**0.022**	9.02 (0.36)
But (N_2_)	17.06 (0.33)	**0.0051**	17.56 (0.10)	**0**	17.26 (1.75)	0.14	NG	NG	19.22 (0.82)	**0.0019**	16.56 (0.96)	0.12	15.4 (0.39)
H_2_ (NH_4_Cl)	94.03 (22.7)	0.1454	84.97 (1.66)	**0.009**	81.14 (1.5)	**0.011**	73.59 (5.47)	0.063	73.57 (10.03)	0.14	89.87 (3.64)	**0.01**	56.25 (3.45)
H_2_ (N_2_)	267.23 (123)	0.21	192 (13.97)	0.103	191.1 (20)	0.143	NG	NG	185.85 (69.6)	0.55	115.83 (27.5)	**0.11**	158.82 (23.3)
Fe(II) (NH_4_Cl)	ND	ND	ND	ND	416.54 (27.27)	0.366	805.62 (165)	**0.0218**	706.5 (13.28)	**0.01**	296.7 (11.71)	0.8	321.34 (159)
Fe(II) (N_2_)	ND	ND	NG	NG	919.89 (146.9)	0.874	NG	NG	714.13 (60.7)	**0.008**	792.07 (194)	0.376	905.17 (33.2)

^*a*^ Growth parameters obtained from butyrate are from 1 mM butyrate. Values are averages from biological triplicates. Standard error values are in parentheses. *P, P* values against wild-type values. *P* values in bold indicate statistical significance. But, butyrate; Fe, iron; NH_4_Cl, ammonium chloride; N_2_, nitrogen gas; NG, no growth; ND, not determined—almost close to the whole growth time. Final OD obtained from 14 days of growth under PE was reported as max OD.

The engineered strain Ω*rub(I&II*) carrying overexpressed RuBisCO form I and II genes did not show any significant differences in growth compared to wild-type strains when grown under non-nitrogen- fixing or under nitrogen-fixing conditions (Table S1; Table 3; Fig. 3C and D).

### Deletion of the *phaR* gene or overexpression of the RuBisCO form I and II genes increases PHA production in TIE-1 under photoheterotrophic growth with butyrate

Deletion of the *phaR* (regulator gene) increased PHA production (mg/L/cell and dry cell weight % wt/wt) ~2X (*P* = 0.00) compared to wild-type under non-nitrogen-fixing or nitrogen-fixing conditions (Fig. 3E; Table 4; Table S2). Deletion of the *phaZ, gly,* or *nifA* genes resulted in an overall decrease in PHA production (mg/L/cell and dry cell weight % wt/wt) of ~10X (*P* = 0.05) compared to wild-type under growth under non-nitrogen-fixing conditions. These deletions did not seem to affect PHA production when compared to wild-type under nitrogen-fixing conditions. We did not detect any PHA from the *ΔnifA* grown under nitrogen-fixing conditions as it did not show any growth. Except for Δ*phaR*, all strains exhibited a consistent increase in PHA production during nitrogen-fixing growth conditions in comparison to growth under non-nitrogen- fixing conditions.

**TABLE 4:** PHA production from different TIE-1 strains under photoautotrophic and heterotrophic growth conditions^*a*^

	Δ*phaR*	*P*	Δ*phaZ*	*P*	Δ*gly*	*P*	Δ*nifA*	*P*	Ω*rub(I*)	*P*	Ω*rub(I&II*)	*P*	WT
PHA (mg/L)													
Butyrate (NH_4_Cl)	30.37 (0.33)	**0.03**	4.86 (0.69)	0.116	5.25 (0.67)	0.098	8.21 (0.3)	0.29	36.03 (1.61)	0.003	41.1 (0.52)	**0.016**	12.061 (0.27)
Butyrate (N_2_)	14.3 (0.46)	**0.003**	13.73 (1.68)	**0.001**	15.87 (1.36)	**0.07**	NG	NG	49.71 (4.88)	0.002	53.23 (2.91)	**0**	25.138 (0.05)
H_2_ (NH_4_Cl)	24.45 (2.08)	0.349	24.51 (1.33)	0.387	29.18 (0.14)	0.754	34.65 (3.1)	0.58	40.15 (4.25)	0.234	32.8 (3.57)	0.819	31.089 (1.37)
H_2_ (N_2_)	32.49 (1.25)	0.505	30.82 (1.21)	0.556	16.09 (0.76)	0.151	NG	NG	32.72 (1.16)	0.655	13.06 (1.16)	0.115	25.757 (0.26)
Fe(II) (NH_4_Cl)	1.25 (0.04)	**0.013**	ND	ND	1.46 (0.04)	0.092	1.7 (0.01)	0.38	1.85 (0.034)	0.374	1.79 (0.075)	0.394	2.19 (0.03)
Fe(II) (N_2_)	0.24 (0.012)	**0**	0.29 (0.02)	0.002	0.42 (0.008)	**0.02**	NG	NG	0.28 (0.018)	0.005	0.80 (0.004)	0.663	0.96 (0.03)
PE (NH_4_Cl)	0.40 (0.46)	**0.0002**	1.23 (0.05)	**0.000**	0.735 (0.18)	**0.000**	0.59 (0.008)	**0.00**	0.62 (0.17)	**0.000**	0.64 (0.037)	**0.000**	2.57 (0.058)
PE (N_2_)	0.422 (0.096)	0.604	0.82 (0.009)	**0.01**	0.59 (0.03)	0.14	NG	NG	0.67 (0.17)	0.14	0.33 (0.012)	0.94	0.34 (0.02)
PHA (mg/L/cell) (x10^−11^)													
Butyrate (NH_4_Cl)	2.32 (0)	**0**	0.079 (0.03)	0.051	0.11 (0.03)	0.056	0.1 (0.03)	0.05	2.91 (0.22)	**0.007**	5.29 (0.1)	**0.001**	1 (0.01)
Butyrate (N_2_)	1.89 (0.07)	**0.02**	1.89 (0.23)	0.606	1.4 (0.12)	0.374	NG	NG	5.16 (0.45)	**0.00**	8.41 (0.47)	**0.00**	2.65 (0.02)
H_2_ (NH_4_Cl)	2.17 (0.14)	0.098	2.63 (0.18)	0.127	3.22 (0.01)	0.056	3.47 (0.27)	**0**	3.58 (0.21)	0.055	3.01 (0.27)	**0.012**	1.52 (0.04)
H_2_ (N_2_)	4.89 (0.22)	0.517	3.84 (0.15)	0.952	1.66 (0.07)	0.098	NG	NG	4.07 (0.18)	0.862	1.74 (0.31)	0.111	3.76 (0.05)
Fe(II) (NH_4_Cl)	2.32 (0.06)	**0**	ND	ND	1.9 (0.05)	**0**	4.37 (0.03)	0.37	3.36 (0.71)	0.033	5.64 (0.26)	0.98	5.62 (0.09)
Fe(II) (N_2_)	0.85 (0.03)	0.082	0.45 (0.04)	**0.004**	0.89 (0.03)	0.075	NG	NG	0.35 (0.038)	0.002	0.68 (0.009)	0.111	1.66 (0.08)
PE (NH_4_Cl)	0.10 (0.02)	**0.006**	0.5 (0.02)	0.803	0.35 (0.09)	0.085	0.25 (0.00)	0.25	0.18 (0.04)	0.01	0.17 (0.006)	**0.005**	0.55 (0.006)
PE (N_2_)	0.124 (0.009)	0.89	0.34 (0.023)	**0.023**	0.49 (0.036)	0.011	NG	NG	0.307 (0.11)	0.05	0.207 (0.007)	**0.05**	0.11 (0.016)
PHA (dry cell weight % wt/wt)													
Butyrate (NH_4_Cl)	5.03 (0.00)	**0**	0.15 (0.05)	**0.05**	0.24 (0.23)	**0.05**	0.22 (0.22)	**0.05**	6.30 (0.48)	**0.007**	11.45 (0.20)	**0.001**	2.15 (0.02)
Butyrate (N_2_)	4.08 (0.15)	**0.02**	4.075 (0.25)	0.606	3.03 (0.26)	0.374	NG	NG	11.18 (0.98)	**0**	18.23 (1.00)	**0**	5.73 (0.03)
H_2_ (NH_4_Cl)	4.70 (0.30)	0.098	5.7 (0.375)	0.127	6.98 (0.02)	0.056	7.53 (0.58)	**0**	7.75 (0.45)	**0.055**	6.52 (0.58)	**0.012**	3.28 (0.08)
H_2_ (N_2_)	10.60 (0.48)	0.517	8.325 (0.32)	0.952	3.57 (0.15)	0.098	NG	NG	8.83 (0.38)	0.862	3.75 (0.50)	0.111	8.15 (0.10)
Fe(II) (NH_4_Cl)	5.03 (0.14)	**0**	ND	ND	4.10 (0.13)	**0**	9.4 8(0.07)	0.37	7.28(1.50)	**0.033**	12.23 (0.58)	0.98	12.20 (0.20)
Fe(II) (N_2_)	1.86 (0.08)	0.082	0.9825 (0.1)	**0.004**	1.93 (0.07)	0.075	NG	NG	0.75 (0.80)	**0.002**	1.48 (0.02)	0.111	3.60 (0.19)
PE (NH_4_Cl)	0.22 (0.03)	**0.006**	1.075 (0.02)	0.803	0.76 (0.18)	0.085	0.53 (0.00)	0.25	0.38 (0.08)	**0.01**	0.35 (0.01)	**0.005**	1.18 (0.018)
PE (N_2_)	0.25 (0.00)	0.89	0.725 (0.02)	0.023	1.05 (0.08)	**0.011**	NG	NG	0.65 (0.24)	**0.05**	0.43 (0.02)	**0.05**	0.24 (0.03)

^*a*^ Values are averages from biological triplicates, except for those from photoelectrotrophic (PE) growth conditions, which are duplicates. PHA from growth on butyrate are from 10 mM concentrations. Standard error values are in parentheses. *P, P* values against wild-type values. *P* values in bold indicate statistical significance. Fe, iron; NH_4_Cl, ammonium chloride; N_2_, nitrogen gas; PE. photoelectrotrophic growth; NG, no growth; ND, not detectable.

When grown photoheterotrophically with butyrate, the two strains harboring the Ω*rub(I*) and Ω*rub(I&II*) demonstrated increased PHA production, regardless of the nitrogen source. Under non-nitrogen-fixing conditions, the Ω*rub(I*) strain had 2X (*P* = 0.00) higher PHA production (mg/L/cell and dry cell weight % wt/wt) than wild-type, while the Ω*rub(I&II*) exhibited a 5X increase (*P* = 0.001). This increase in PHA was also observed under nitrogen-fixing conditions where an increase of 1.9X (*P* = 0.007) was observed from the Ω*rub(I*) and 3X (*P* = 0.001) from the Ω*rub(I&II)* (Fig. 3F; Table 4; Table S2). Like the deletion mutants, an overall increase in PHA production was observed from the engineered and wild-type strains under nitrogen-fixing vs non-nitrogen-fixing conditions (Fig. 3F).

### Deletion of the *phaR*, *phaZ,* or overexpression of RuBisCO form I and II genes increased the final optical density of TIE-1 under photoautotrophic growth with H_2_ under non-nitrogen-fixing conditions

**Fig 4:**
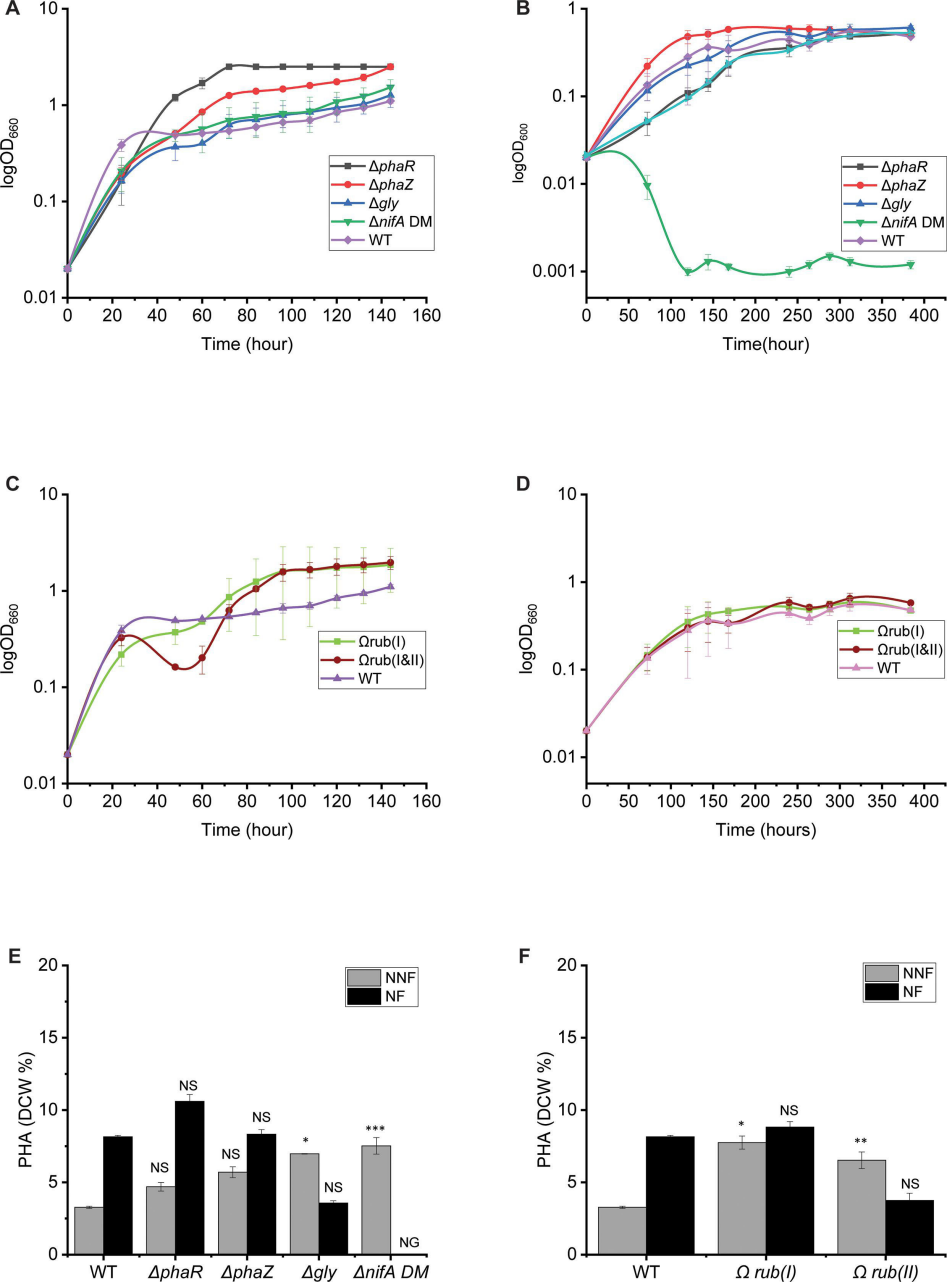
Growth and PHA production (dry cell weight % wt/wt) from different strains grown with fresh water basal media with hydrogen. (A) Growth of all the mutant strains under non-nitrogen-fixing conditions. (B) Growth of all the mutant strains under nitrogen-fixing conditions. (C) Growth of the engineered RuBisCO strains under non-nitrogen-fixing conditions. (D) Growth of all the engineered RuBisCO stains under nitrogen-fixing conditions. (E) PHA production from mutants and TIE-1 wild-type strains grown under non-nitrogen-fixing or nitrogen-fixing conditions. (F) PHA production from Rubisco engineered and wild-type strains grown under hydrogen non-nitrogen-fixing or nitrogen-fixing conditions. NFF, non-nitrogen fixing; NN, nitrogen fixing. Error bars from growth curves represent the standard deviation calculated from three replicates, while error bars from PHA production depict the standard error derived from biological triplicates. The statistical differences in PHA production were calculated using two-tailed Student’s test against the wild-type TIE-1. *P ≤ 0.05, **P < 0.01, and ***P < 0.001; NS, not significant; ND, not detected; NG, no growth. Δ*phaR* reached the maximum OD 3.7 X faster than wild-type (*P* = 0.00) (whereas the Δ*phaZ* mutant reached maximum OD almost 50 hours earlier than wild-type (*P* = 0.00). Δ*gly* and Δ*nifA* showed similar growth patterns as wild-type under growth with H_2_ and non-nitrogen-fixing conditions (Fig. 4A). No significant difference was observed under nitrogen-fixing conditions with hydrogen from all the mutants, except the Δ*nifA,* which showed no growth as shown in Table 3; Fig. 4B.

Achieving production from a low-cost and abundant carbon source is one of the most impactful pathways for bioplastic production. Accordingly, we tested the growth of all the strains under photoautotrophic conditions using H_2_ as an electron source and CO_2_ as a carbon source in a freshwater basal liquid medium. Light is used as the energy source as TIE-1 is a photosynthetic bacterium. Fig. 4A; Table 3 show that although Δ*phaR* and Δ*phaZ* mutants showed a slightly longer generation time (~3 hours slower) (*P* = 0.001) , they reached the maximum OD faster than wild-type strains when grown with H_2_ under non-nitrogen-fixing conditions.

Although the strains Ω*rub(I&II*) showed an extended lag time (1.6 X slower than wild-type *P* = 0.01), they reached a higher final OD (0.52 higher) than wild-type (Table 3) when grown with H_2_ and under non-nitrogen-fixing conditions. The Ω*rub(I*) did not show any significant growth difference compared to wild-type (Table 3; Fig. 4C). No difference was observed between the two engineered strains when grown with H_2_ under nitrogen-fixing conditions (Table 3; Fig. 4D).

### Deletion of the glycogen synthase (*gly*), *nifA* genes, or overexpression of RuBisCO form I or I & II increased PHA production under photoautotrophic growth with H_2_ under non-nitrogen-fixing conditions

We tested PHA production (mg/L/cell and dry cell weight % wt/wt) from all the constructed strains under photoautotrophic growth conditions with H_2_. The mutants Δ*phaR* and Δ*phaZ* did not show any significant difference in PHA production compared to wild-type under growth with H_2_ regardless of the nitrogen source. The Δ*gly* and the Δ*nifA* strains showed a 2X increase (*P* = 0.05 and *P* = 0, respectively) in PHA production when grown with H_2_ under non-nitrogen-fixing conditions (Table 4; Fig. 4E). The deletion of *phaR* or *phaZ* did not affect PHA production compared to wild-type strains. No PHA was produced by the Δ*nifA* during growth under nitrogen-fixing conditions due to its inability to grow. Unlike growth with butyrate, switching from non-nitrogen-fixing to nitrogen-fixing conditions increased PHA production only in wild-type TIE-1 but not in the mutants. The mutants showed lower PHA production compared to wild-type under nitrogen-fixing conditions with H_2_ as an electron source.

Like growth with butyrate, both engineered Ω*rub(I*) and Ω*rub(I*&*II*) strains showed an increase in PHA production (mg/L/cell and dry cell weight % wt/wt) when grown with H_2_ under non-nitrogen-fixing conditions. PHA production was nearly double in both engineered strains compared to wild-type (*P* = 0.05 and 0.01, respectively), (Table 4; Fig. 4F). Switching to nitrogen-fixing conditions with H_2_ did not affect PHA production (mg/L/cell and dry cell weight % wt/wt) of the two engineered strains Ω*rub(I*) and when compared to wild-type (Table 4; Fig. 4F). In contrast, a decrease in PHA production was observed from the Ω*rub(I&II*) under nitrogen-fixing conditions (Table 4; Fig. 4F).

### Deletion of the *phaR* and *phaZ* genes impaired the ability of TIE-1 to oxidize Fe(II)

**Fig 5:**
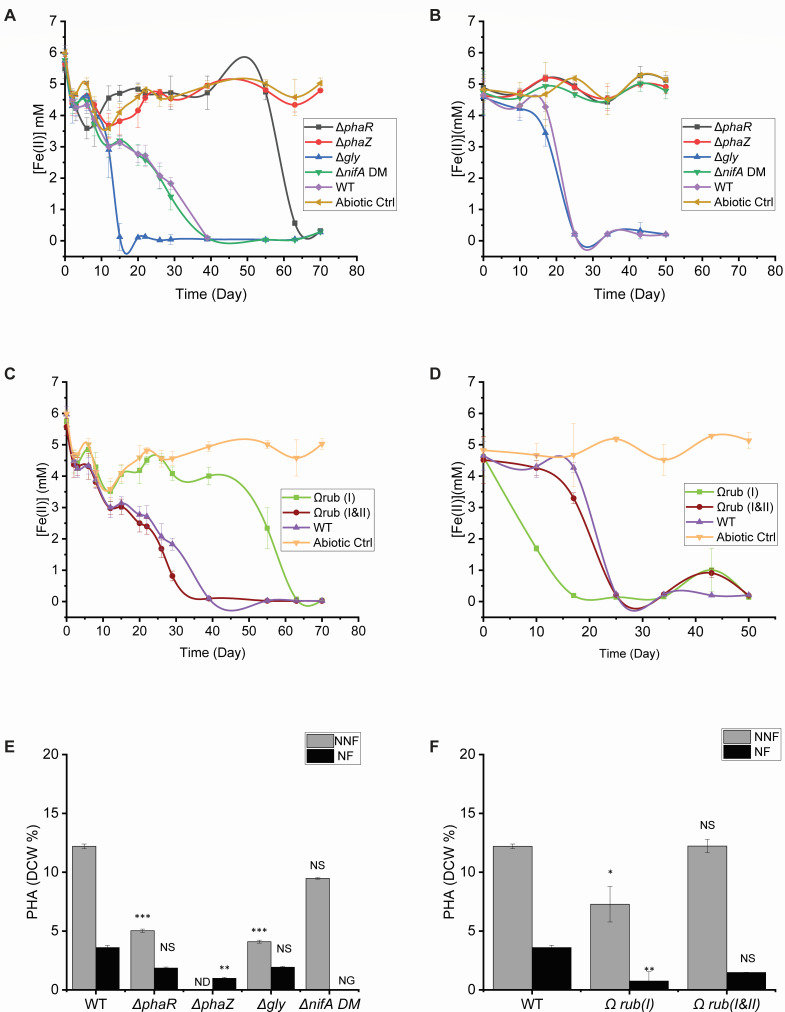
Growth and PHA production (dry cell weight % wt/wt) from strains grown under photoferrotrophy. (A) Fe(II) concentration variation from the mutants grown under non-nitrogen-fixing conditions. (B) Fe(II) concentration variation from the mutans during nitrogen-fixing conditions with N_2_. (C) Growth of the engineered RuBisCO strains under non-nitrogen-fixing conditions. (D) Growth of all the engineered RuBisCO stains under nitrogen-fixing growth conditions. (E) PHA productivity from mutants grown under non-nitrogen-fixing conditions photoferrotrophically. (F) PHA production from engineered RuBisCO strains grown under non-nitrogen-fixing or nitrogen-fixing conditions. Error bars from Fe(II) oxidation curves represent the standard deviation calculated from three replicates, while error bars from PHA production depict the standard error derived from biological triplicates. NFF, non-nitrogenfixing; NN, nitrogen fixing. The statistical differences in PHA production were calculated using two-tailed Student’s test against the wild-type TIE-1. *P ≤ 0.05, **P < 0.01, and ***P < 0.001; NS, not significant; ND, not detected; NG, no growth.

TIE-1 can use electrons produced by the oxidation of Fe(II) for photoautotrophy (photoferrotrophy). Under these difficult growth conditions, the main carbon source is CO_2_ and the energy source is light. We tested the growth of all the strains we constructed under photoferrotrophic growth conditions. All the strains were first pre-grown in H_2_ to allow the expression of the genes involved in Fe(II) oxidation in TIE-1, as performed previously (27). We observed a defect in the ability of TIE-1 to oxidize Fe(II) and grow when the *phaZ* or *phaR* genes were deleted (Fig. 5A and B). Under non-nitrogen-fixing conditions, the *phaZ* mutant was not able to oxidize Fe(II) or grow even after 50 days (*P* = 0.003 at day 50 compared to the wild-type). A significant delay in Fe(II) oxidation was observed in the *phaR* mutant under non-nitrogen-fixing conditions. Δ*phaR* was able to fully oxidize Fe(II) only after 65 days versus 40 days (*P* < 0.001 at day 40) for wild-type and grow to wild-type levels. In contrast, Δ*gly* showed faster Fe(II) oxidation ability, which occurred after 15 days of growth (*<*0.001 at day 15) as opposed to ~40 days for wild-type when grown under non-nitrogen-fixing conditions. Δ*nifA* showed a similar iron oxidation pattern as wild-type under non-nitrogen-fixing conditions (Fig. 5A). Under non-nitrogen-fixing conditions, the oxidation ability of Δ*gly* was similar to that of the wild-type. As expected, no Fe(II) oxidation occurred from Δ*nifA* as the strain was not able to grow under nitrogen-fixing conditions. Overexpression of RuBisCO form I appears to delay the ability of TIE-1 to oxidize Fe(II) under non-nitrogen-fixing conditions. However, when RuBisCO form I and II genes are both overexpressed, the strain oxidizes Fe(II) at the same time as wild-type (~40 days) (Fig. 5C). During nitrogen fixation, the Ω*rub(I*) showed faster Fe(II) oxidation compared to wild-type (*P* < 0.001 at ~day 30), whereas the Ω*rub(I&II*) initiated Fe(II) oxidation at the same time as wild-type (*P* = 0.42 at ~day 30), (Fig. 5D).

### Deletion of the various genes or overexpression of RuBisCO form I and II genes did not improve PHA production in TIE-1 under photoferrotrophy

To test PHA production obtained from growth during photoferrotrophy, samples were collected right after complete Fe(II) oxidation occurred. Lower PHA production (mg/L/cell and dry cell weight % wt/wt) was observed across all the mutants under growth with Fe(II) and non-nitrogen-fixing conditions compared to wild-type (Table 4; Fig. 5E). Among all the mutants, the Δ*nifA* had the highest PHA production when grown under non-nitrogen-fixing conditions, with comparable levels to wild-type (*P* = 0.37).

Δ*phaR* and Δ*gly* showed the lowest production, about half that observed in wild-type when grown under non-nitrogen-fixing conditions (*P* = 0). No PHA was detected from the Δ*phaZ* mutant from the non-nitrogen-fixing conditions, which is linked to its inability to oxidize Fe(II). Like growth under non-nitrogen-fixing conditions, PHA productions from the Δ*phaR*, Δ*phaZ,* and Δ*gly* (*P* = 0) strains during nitrogen-fixing conditions were overall lower than that obtained from wild-type (Fig. 5E and F). PHA production (mg/L/cell and dry cell weight % wt/wt) obtained from Δ*pha*z under nitrogen-fixing conditions was ~3X (*P* = 0.004) smaller than the wild-type (Table 4; Fig. 5E). No PHA was obtained from Δ*nifA* when grown under nitrogen-fixing conditions because this strain is incapable of growth under such conditions.

A decrease in PHA production of about half was observed from the single engineered Ω*rub(I)* strain under Fe(II) under non-nitrogen-fixing growth conditions (*P* = 0.033) (Table 4; Fig. 5F). However, PHA production obtained from the Ω*rub(I&II)* was similar to the wild-type under growth with Fe(II) and non-nitrogen-fixing conditions (*P* = 0.98). Like the trend obtained from the mutants, a decrease in PHA production was observed in the RuBisCO overexpressing strains under nitrogen-fixing conditions compared to the growth under non-nitrogen-fixing conditions. PHA production obtained from the Ω*rub(I)* was ~4X (*P* = 0.002) less than what was obtained from wild-type under nitrogen-fixing conditions.

### Growth under photoelectrotrophy under non-nitrogen-fixing conditions showed increased electron uptake from the engineered RuBisCO strains

In addition to its ability to grow autotrophically using hydrogen and Fe(II) as electron sources, TIE-1 can uptake electrons directly from poised electrodes (32). We grew the cells in bioelectrochemical reactors as described in Materials and Methods with freshwater media under nitrogen-fixing or non-nitrogen-fixing conditions. All mutant strains except Δ*phaR* showed longer generation time compared to wild-type strains when grown under non-nitrogen-fixing conditions (Table 3) (*P* < 0.05). Δ*phaZ* and Δ*gly* exhibited significantly slower growth, as evidenced by a generation time ~4X longer than that of wild-type. Δ*nifA* similarly exhibited an extended generation time, ~3X longer than that of wild-type (Table 3) (*P* = 0.0001). Δ*phaR* did not exhibit a significant difference in its generation time compared to wild-type when grown under non-nitrogen-fixing conditions. *Ωrub(I)* and *Ωrub(I&II)* did not show a defect in generation time compared to wild-type under photoelectrotrophic non-nitrogen-fixing conditions.

**Fig 6:**
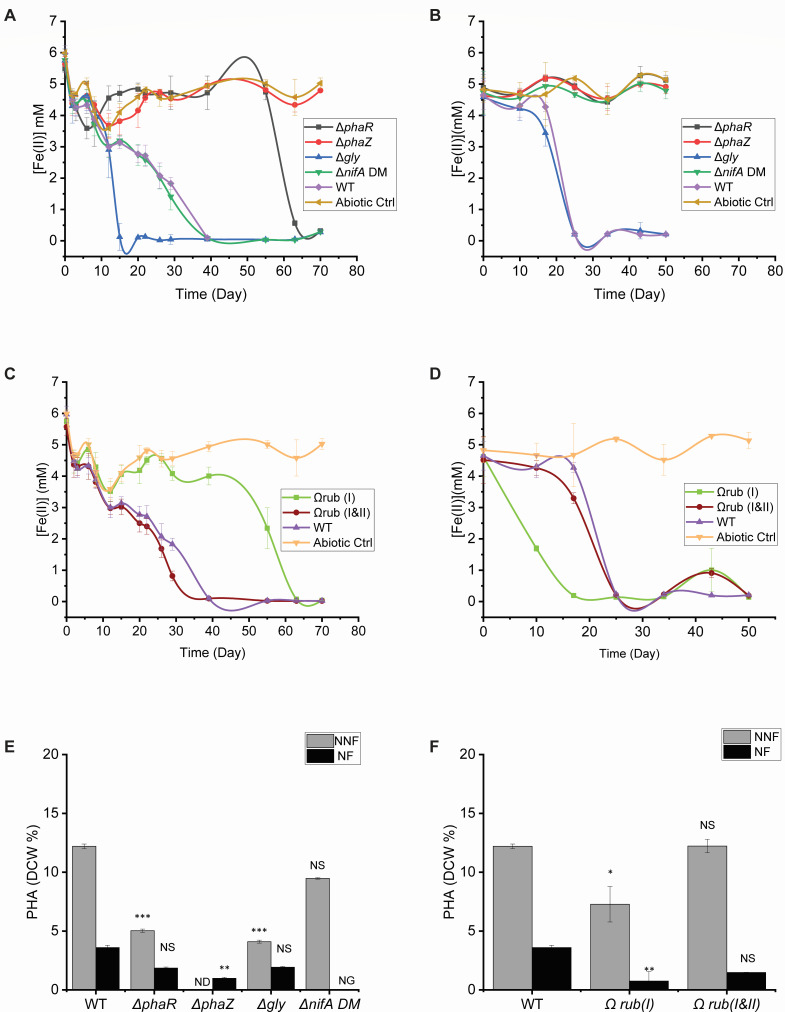
Electron uptake and PHA production under EEU. (A and B) Current density and current uptake, respectively, from the wild-type TIE-1 (WT) strain, the various mutants Δ*phaR*, Δ*phaZ*, Δ*gly*, and Δ*nifA* DM (double mutant), and the engineered strains Ω*rub(I)* and Ω*rub(I&II)* under non-nitrogen-fixing growth conditions. (C and D) Current density and current uptake, respectively, from the wild-type TIE-1 (WT) strain, the various mutants Δ*phaR*, Δ*phaZ*, Δ*gly*, and Δ*nifA* DM (double mutant), and the engineered strains Ω*rub(I)* and Ω*rub(I&II)* under nitrogen fixation conditions (N_2_). (E) PHA production (dry cell weight % wt/wt) from mutants grown under non-nitrogen-fixing and nitrogen-fixing conditions photoelectrotrophically. (F) PHA productivity from engineered RuBisCO strains grown under photoelectrotrophic non-nitrogen-fixing and nitrogen-fixing growth conditions. The statistical differences in PHA production were calculated using two-tailed Student’s test against the wild-type TIE-1. *P ≤ 0.05, **P < 0.01, and ***P < 0.001 NNF, non-nitrogen fixing; NF, nitrogen fixing. Error bars represent standard errors from biological duplicates.

We also measured the final OD of the strains at the end of the 14-day incubation. These values are reported as max OD in Table 3. The final OD values were ~1/2 lower than those of wild-type from Δ*phaZ*, Δ*gly*, and Δ*nifA* under non-nitrogen-fixing growth conditions (*P* < 0.05). *ΔphaR* as well as the engineered strains *Ωrub(I)* and *Ωrub(I&II)* had the same final OD as wild-type (Table 3) when grown under non-nitrogen-fixing conditions. Under photoelectrotrophic and nitrogen-fixing conditions, the generation time of Δ*phaR,* Δ*phaZ*, as well as *Ωrub(I)* and *Ωrub(I&II)* did not show any significant differences compared to wild-type (Table 1). The Δ*nifA* did not show growth as expected. Due to the intricate nature of the experimental setup in photoelectrotrophic growth, we could only measure the cell density at initial and final times, preventing us from reporting lag time. We then assessed the ability of each constructed strain to capture electrons from a poised electrode. Under non-nitrogen-fixing conditions, Δ*pha*R, Δ*phaZ*, Δ*nifA*, and *Ωrub(I)* did not show a significant difference in current uptake when compared to wild-type. However, Δ*gly* showed ~6X (*P* = 0.001) lower current uptake, while *Ωrub(I&II)* showed a decrease of ~2X (*P* = 0.009) in electron uptake when compared to wild-type (Fig. 6; Table 5).

**TABLE 5:** Current uptake obtained from the different strains during photoelectroautotrophic growth under non-nitrogen-fixing or nitrogen-fixing conditions^*a*^

Strain	Current uptake (C)	
	Growth under non-nitrogen-fixing conditions	Growth under nitrogen-fixing conditions
WT TIE-1	−0.7891 ± 0.02	−1.2359 ± 0.1027
Δ*pha*R	−0.5257 ± 0.2167 (*P* = 0.00018)	−0.0666 ± 0.2182 (*P* = 0.0017)
Δ*pha*Z	−0.6311 ± 0.2118 (*P* = 0.0029)	−0.3019 ± 0.1356 (*P* = 0.00092)
Δ*gly*	−0.1241 ± 0.0015 (*P* = 0.0001)	−0.4695 ± 0.014 (*P* = 0.00036)
Δ*nifA*	−0.8461 ± 0.0595 (*P* = 0.00026)	−0.0938 ± 0.0356 (*P* = 0.00045)
Ω*rub(I)*	−0.9922 ± 0.014 (*P* = 0.00177)	−5.6128 ± 0.1182 (*P* = 0.00332)
Ω*rub(I &II)*	−0.3513 ± 0.0283 (*P* = 0.0009)	−20.2604 ± 0.2492 (*P* = 0.0008)
Abiotic control	−0.1159 ± 0.067 (*P* = 0.00021)	−0.1474 ± 0.0741 (*P* = 0.00067)

^*a*^ Values are from two biological replicates, ± standard deviation values.

Under nitrogen fixation conditions, electron uptake varied among strains. Notably, Δ*phaR* exhibited an 18X reduction compared to wild-type (*P* = 0.001). Additionally, both Δ*phaZ* and Δgly displayed 4- and 2.6X lower uptake, respectively (*P* < 0.001). The Δ*nifA*, which expectedly was unable to grow under nitrogen fixation conditions, demonstrated an electron uptake like that of abiotic control. In contrast, *Ωrub(I)* and *Ωrub(I&II)* displayed 4.5 and 16X higher electron uptake than wild-type, respectively (Table 5) (*P* < 0.005).

### Overexpressing the RuBisCO form I and form II of TIE-1 increased its PHA production under photoelectrotrophic nitrogen-fixing growth conditions but not under non-nitrogen-fixing conditions

When grown under non-nitrogen-fixing conditions, PHA values obtained from the wild-type are sufficiently low so as to hinder us from comparing between strains (Table 4; Fig. 6E and F).

When grown under nitrogen-fixing conditions, we observed that the PHA production of *phaR* is similar to that of wild type. However, PHA production increased by 3X in Δ*phaZ* and Δ*gly* compared to wild-type (Table 4; Fig. 6E) (*P* < 0.05). PHA production by *Ωrub(I) and Ωrub(I&II)* went 2X when compared to wild-type (*P* = 0.05). However, under nitrogen-fixing conditions, PHA produced from *Ωrub(I)*= 0.24% and *Ωrub(I&II)* =0.16% (dry cell weight % wt/wt) did not show statistically significant differences (*P* = 0.21) when evaluated against each other.

## DISCUSSION

In this study, we demonstrated that TIE-1 can be modified using genetic engineering and synthetic biology tools to enhance polyhydroxyalkanoate (PHA) production. We found that deleting the *phaR* regulator gene of PHA biosynthesis increased PHA production under non-nitrogen-fixing conditions with butyrate. Deletion of the glycogen synthase and the regulators of the nitrogen fixation pathways *nifA_1_* and *nifA_2_* increased PHA accumulation when grown under non-nitrogen-fixing conditions with hydrogen. More importantly, overexpressing RuBisCO form I and II using the φC31 integrase system in TIE-1 enhanced PHA production under photoheterotrophic growth with butyrate, regardless of the nitrogen source. This was also the case for autotrophic growth of these TIE-1 strains with hydrogen in non-nitrogen-fixing conditions. These results indicate that genetic engineering techniques, including our newly developed φC31 integration system, are effective for genetic manipulation in TIE-1 and increasing PHA production.

### Effect of deletions of genes in the PHA pathway on growth and PHA production in TIE-1

The specific regulatory role of PhaR (Rpal_0531) has not been explored in TIE-1 before. Here, we discovered that deleting *phaR* did not significantly affect PHA production in TIE-1 across all growth conditions tested. However, the Δ*phaR* mutant showed growth defects under non-nitrogen-fixing photoheterotrophic growth with butyrate and photoautotrophic growth with H_2_ (Table S1; Table 3). Additionally, it exhibited decreased Fe(II) oxidation and electron uptake under nitrogen-fixing conditions compared to the wild-type (Fig. 5). In *Bradyrhizobium diazoefficiens* USDA110, inactivation of *phaR* decreased PHA accumulation and affected processes such as exopolysaccharide biosynthesis and heat stress tolerance (67). Our results suggest that PhaR’s role in TIE-1 may extend to other pathways. Further transcriptomic and proteomic analyses of this mutant are needed to understand PhaR’s broader regulatory roles.

**Fig 7:**
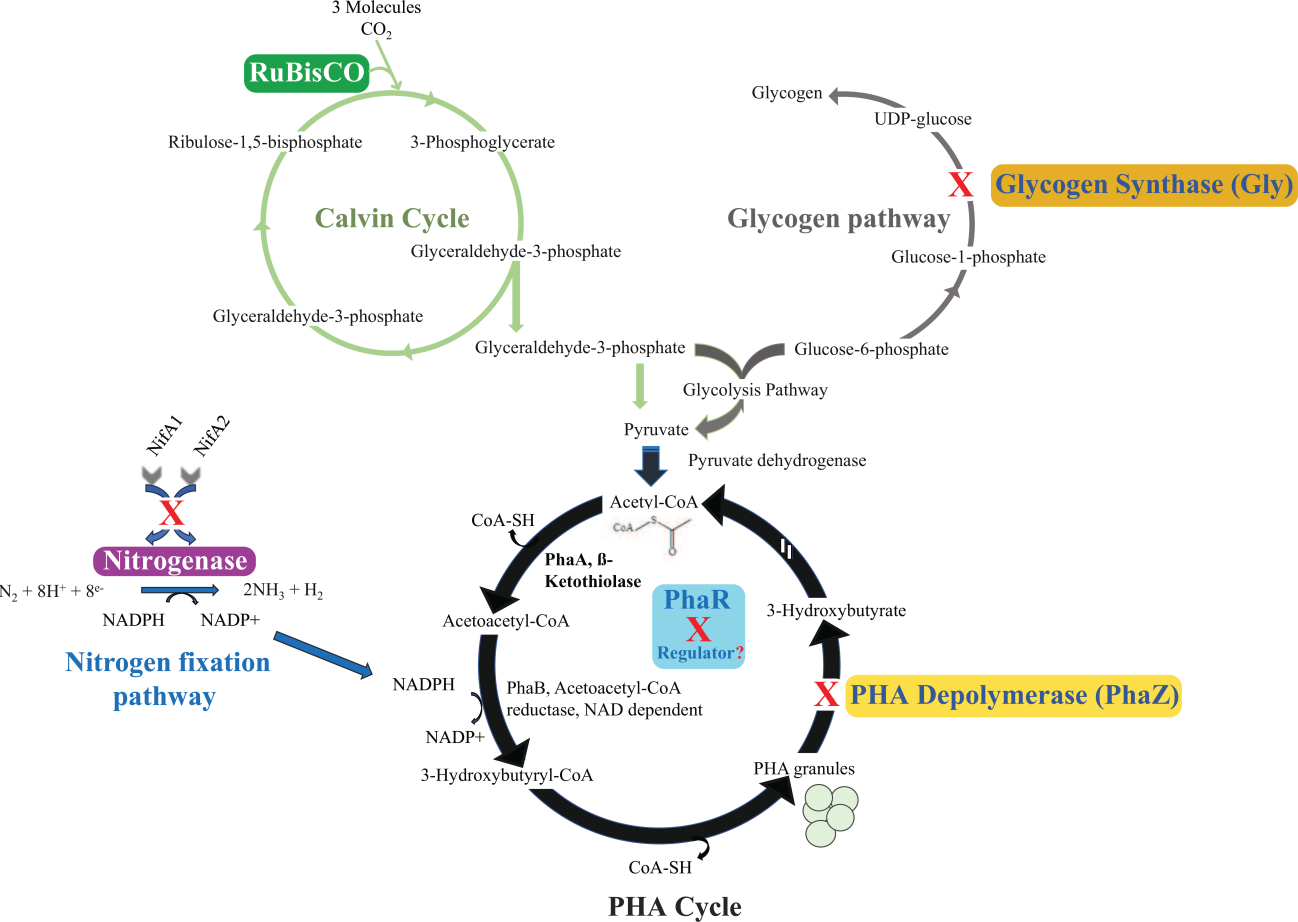
Summary of pathways targeted to increase PHA production in TIE-1. (**A**) Calvin–Benson–Bassham cycle: overexpression of RuBisCo forms I and II. (**B**) Nitrogen fixation pathway: deletion of the two regulators *nifA1* and *nifA2* gene. (**C**) Glycogen pathway: deletion of the glycogen synthase gene. (**D**) PHA biosynthesis pathway: deletion of the PHA depolymerease *phaZ* and the regulator *phaR* genes. Red X represents deletion.

We hypothesized that deleting the PHA depolymerase (PhaZ) in TIE-1 would inhibit PHA degradation, potentially increasing total PHA storage (Fig. 7). This expectation was based on findings from other purple nonsulfur bacteria, such as *Rhodobacter sphaeroides*, where the *phaZ* mutant showed improved PHA accumulation under nitrogen-rich conditions (68). However, deleting *phaZ* did not significantly elevate PHA production in TIE-1. Unexpectedly, the absence of *phaZ* affected TIE-1′s ability to oxidize Fe(II) during photoferrotrophic growth and decreased electron uptake under nitrogen-fixing photoelectrotrophic growth conditions. These findings suggest that disrupting PHA depolymerase alters the electron balance within TIE-1, meriting further bioinformatics and proteomics studies.

### Effects of deletion of a potential competitive pathway on PHA production in TIE-1

We hypothesized that deleting the glycogen synthase (*gly*) gene would prevent glucose accumulation as glycogen, allowing glucose to be converted into pyruvate via glycolysis and subsequently into acetyl-CoA by pyruvate dehydrogenase, as illustrated in Fig. 7. Increased availability of acetyl-CoA could enhance PHA production. Contrary to our expectations, deletion of the glycogen synthase gene increased PHA production only under non-nitrogen-fixing conditions with hydrogen. A decrease in PHA production was observed under photoheterotrophic growth with butyrate and photoautotrophic growth with Fe(II) under nitrogen-fixing growth conditions. Additionally, a shorter generation time under non-nitrogen-fixing conditions with butyrate and Fe(II) suggests that blocking glycogen accumulation might channel carbon into biomass production rather than PHA production. Similar findings were reported in *Synechocystis sp.* PCC 6803, where glycogen was hypothesized to serve as a carbon source for PHA synthesis under nitrogen starvation conditions (58). Although autotrophic growth with hydrogen could be considered stressful compared to photoheterotrophic growth, the Δ*gly* strain seems to prioritize biomass increase over PHA production under these conditions.

### Effect of nitrogen fixation and *nifA* gene deletion on PHA production in TIE-1

Nitrogen fixation has been reported to increase PHA production (27, 41, 61). We observed this trend under photoheterotrophic growth with butyrate and photoautotrophic growth with hydrogen across most strains. However, under photoautotrophic growth with Fe(II), PHA production decreased under nitrogen-fixing compared to non-nitrogen-fixing conditions, with similar values obtained from photoelectrotrophic conditions under the two nitrogen sources. These observations align with those of our previous studies (27). The distinct responses under different growth conditions suggest potential variation in the regulatory mechanisms employed by TIE-1 under nitrogen fixation conditions.

Nitrogen fixation requires substantial reducing power (37). We investigated the impact of deleting two key activators of the nitrogenase genes, *nifA_1_* and *nifA_2_*, to redirect NADPH toward PHA biosynthesis (Fig. 7). Contrary to our expectation, deleting *nifA* genes mainly decreased PHA production. However, under non-nitrogen-fixing photoheterotrophic growth with 10 mM butyrate, this strain showed a decreased generation time by approximately 25% (*P* = 0.007, Table 1; Table S2) suggesting that it favored growth over PHA production. Due to the highly reduced nature of butyrate and the presence of sodium bicarbonate in our media, it is possible that the electrons intended for nitrogen fixation were instead diverted to CO_2_ fixation for redox balance. This diversion could have contributed to biomass production rather than PHA synthesis. It is worth mentioning though that like the increase in *n*-butanol production observed in Δ*nifA* (37), we noticed an increase in PHA production from this strain grown under non-nitrogen-fixing photoautotrophic conditions with hydrogen (37).

### Effect of overexpressing RuBisCO genes on PHA production in TIE-1

By increasing CO_2_ fixation through the Calvin–Benson–Bassham, we aim to increase acetyl-CoA abundance, leading to higher PHA production as shown in Fig. 7. Similar observations were reported in *Ralstonia eutropha*, where expressing a heterologous cyanobacterial RuBisCO gene resulted in a significant 99.7% increase in PHA accumulation under autotrophic growth. In TIE-1, overexpression of RuBisCO form I led to a ~ 2X increase in PHA production under both non-nitrogen- and nitrogen-fixing conditions. Overexpression of both RuBisCO forms I and II resulted in a ~ 5X increase under photoheterotrophic growth with butyrate and a ~ 3X increase under nitrogen-fixing conditions. The combined overexpression of both forms had a cumulative effect on PHA production. Surprisingly, overexpression of RuBisCO form I alone showed a growth defect under photoheterotrophy with butyrate and impaired Fe(II) oxidation under non-nitrogen-fixing conditions. This defect disappeared when both RuBisCO forms were overexpressed, suggesting a compensatory effect.

### Future directions

We conclude that overexpressing RuBisCO form I and II increased PHA production in TIE-1 more efficiently than deleting competitive pathways. These (*ΩrubI*) and *Ωrub(I&II)* engineered strains could be envisioned as potential candidates for PHA production in larger scale, both under heterotrophic and autotrophic growth conditions. Larger reactors like those developed for biodiesel production by Awogbemi *et al*. (69) could be envisioned for these purposes. Using the φC31 system, it would be highly valuable to investigate the effect of overexpressing other genes that could potentially enhance PHA production in TIE-1. Given that the deletion of *phaR*, glycogen synthase (*gly*), and *nifA* genes did not result in a consistent increase in PHA production, it would be intriguing to initiate overexpression experiments with each of these genes. Additionally, gene overexpression could be extended to the PHA polymerase (*phaC*) gene using the integration system developed in this study to further boost PHA production in TIE-1.

Furthermore, it would be beneficial to assess the effects of creating combinatorial strains by overexpressing *phaR*, *phaZ*, glycogen synthase, and the *nifA* regulator genes, alongside the simultaneous overexpression of both native and non-native RuBisCO form I and II genes in TIE-1. This combinatorial approach may provide deeper insights into the regulatory networks and metabolic pathways influencing PHA synthesis in this organism. We also suggest that metabolic flux balance analysis and other machine learning methods might help us determine optimal mutant and overexpression strain construction for maximizing PHA production.

## MATERIALS AND METHODS

### Bacterial strains, media, and growth conditions

Table 1 lists all the strains used in the study. Lysogeny broth (LB) was used for growth of all *E. coli* strains at 37°C. *Rhodopseudomonas palustris* TIE-1 was grown in the medium containing 3 g/L yeast extract, 3 g/L peptone, 10 mM MOPS [3 N (morpholino) propanesulfonic acid] (pH 7.0), and 10 mM succinate (YPSMOPS) at 30°C. LB and YPSMOPS agar plates were prepared with the addition of 15 g/L agar. When needed, an antibiotic or sucrose was added as indicated in Table S3. All *E. coli* strains were grown on lysogeny broth (LB) at 37°C. Table 1 contains a list of the strains used in the study. Table S3 shows the concentration of antibiotics used as positive and negative selection components.

Anerobic growth of TIE-1 with hydrogen, Fe(II), or using poised electrodes was performed as previously described (27). Because transitioning between heterotrophic and photoautotrophic growth conditions in H_2_ and Fe(II) requires a metabolic shift in TIE-1 (27), to obtain stable growth, cells were first pre-grown in the yeast extract and then transferred into freshwater basal medium with H_2_ for an additional pre-growth before the growth study (27). For growth under photoelectrotrophy, the cells were grown with YP, washed three times with freshwater media, and used directly to innoculate the reactors to reduce contamination. The bioelectrochemical systems were set up as previously described (27) with the electrode modified to carbon felt (dimension 1 × 1×.5 cm).

### Plasmid construction

All the plasmids used in this study are listed in Table 2. The kanamycin and chloramphenicol gene sequences were PCR-amplified from pSRKKm and pSRKCm, respectively. All these antibiotic resistance marker genes were then cloned into the pJQ200KS plasmid separately to replace the gentamicin resistance gene, resulting in pWB091 and pWB092. All the primers used are listed in Table S4.

### Construction of engineered TIE-1 overexpressing RuBisCO form I and form II

Strains were constructed as described previously using markerless integration (34). pWB107 and pWB108 were individually conjugated into TIE-1 through *E. coli* S17-1/λ. After two successive homologous recombination processes, successful integrants were screened by the PCR, as shown in Fig. S4 and S5.

### TIE-1 electroporation

To prepare electrocompetent cells, the TIE-1 strain was inoculated in 500 mL YPSMOPS and then incubated at 30°C. After reaching an OD_660_ of 0.5–0.6, the culture was centrifuged and washed at 4°C at 4,000 X *g* for 10 minutes. After washing five times with 10% glycerol, the cell pellet was resuspended in 2 mL of ice-cold 10% glycerol. This resuspension was aliquoted by 50 µL per sample and then saved in −80°C. For every electroporation, 0.2 µg of plasmid was added to 50 µL thawed electrocompetent cells and mixed well. This mixture was added to 1-mm gap electroporation cuvettes, and then cells were electroporated at 1.8 kV using a Biorad gene Micropulser. After electroporation, the mixture was added to 2 mL warmed Super Optimal Broth (SOB) or LB and grown for 45 minutes. Ten microliters, 100 µL, 500 µL, and the remainder of the culture were plated on the selective medium. Because these plates were further imaged by Nikon A1, glass Petri dishes were used instead of plastic Petri dishes.

### Imaging of *mCherry*

After 4 to 5 days of incubation, the plated electroporated cultures expressing *mCherry* were imaged using the Nikon A1 confocal Eclipse T*i*2 Microscope. For each plate, an image of the whole plate was captured using a camera through both the brightfield and the Texas-red channel (excitation 586 nm; emission 603 nm) with a 10X objective. The colony number of each plate was quantified using NIS-Elements AR Analysis 5.11.01 64-bit software.

### Calculations of transformation efficiency and colony-forming unit per optical density

We evaluated our φC31 phage integration systems by calculating the transformation efficiency as calculated by the following equation:




$$\text{Transformation efficiency}=\frac{\text{Colony number post-electroporation}}{\text{amount of plasmid}\;(\mu g)}$$

The editing efficiency is calculated by:


$$Editing\;efficiency=\;\frac{Number\;of\;engineered\;colonies\;}{Number\;of\;initial\;colonies}$$

### Construction of TIE-1 mutants

The following strains were constructed using the markerless deletion method, as illustrated in Fig. S1: *phaZ* mutant Δ*Rpal_0578*, and *phaR* mutant (Δ*rpal_0531*). The two mutants Δ*gly* and the Δ*nifA* double mutant have been described previously (37). The mutant constructions were as follows: 1 kb upstream and 1 kb downstream of each of the genes of interest were PCR-amplified and cloned into the cloning vector pJQ200KS. The constructed plasmids were then electroporated into the donor strain *E. coli* S17-1/λ. The *E. coli* donor strains carrying the constructed plasmids were conjugated with the TIE-1 wild-type. After two consecutive homologous recombination events, successful mutant candidates were screened using the PCR (Fig. S2) and the primers listed in Table S5.

### PHA extraction and analysis

PHA samples were extracted from 5 mL of the liquid bacterial culture, and the organic phases were analyzed using LC-MS using a method we developed as previously reported (27). About 325 µL of methanol, 400 µL chloroform, and 75 µL of sulfuric acid were simultaneously added to the sample pellet. The mixtures were incubated at 95°C for only 1 hour to minimize the potential loss of methylated products in the aqueous phase. Subsequently, phase separation and acid elimination were performed by the addition of 500 µL of LC-MS-grade water. The organic phases were dried using a speed vacuum. Dried crotonic acid was then resuspended in 50% acetonitrile/50% water and analyzed using an Agilent Technologies 6420 Triple Quad LC/MS. Crotonic acid was detected with a mass-to-charge ratio (*m/z*) of 87. Standard curves of 1, 10, 50, and 100 ppm concentrations were created from a polyhydroxybutyrate (PHB) standard purchased from Sigma Aldrich. Standards were prepared the same way as the samples and were used for the determination of PHA from different samples.

PHA (mg/L) concentrations were determined using the standard curve generated from the counts obtained from the following dry powder PHB concentrations: 1, 10, 50, and 100 ppm.

PHA (mg/L/Cell) was obtained from the following equation:


$$\text{ PHA }(mg/L/\text{ Cell })=\frac{\text{ PHA }(mg/L)}{\text{ Cell number }}$$

PHA productivity in (mg/L/cell/hour) was obtained from the following equation:


$$PHA\;productivity=\frac{\frac{PHA}{cell\;number}}{growth\;time}$$

### OD measurements, cell counts, and cell volume determinations

OD measurements were performed using a Spectronic 200 (Thermo Fisher Scientifc, USA) at 660 nm. Cell enumerations were performed and have been used previously to report PHA production (27, 70). The cell volume was determined by staining TIE-1 with FM 1–43FX membrane stain and visualized under a Nikon, A1 confocal microscope Model Eclipse T*i*2. Stained cells were 3-D-imaged using the FM 1–43 laser channel at 471 nm. Cells were stained according to the manufacturer’s recommendations with a slight modification. Briefly, 1 mL of the culture cell of OD_660_ ~1 was spun down at 15 k rpm for 1 minute, washed once with 1X PBS, and resuspended in 100 µL of ice-cold methanol for 2 minutes. An additional washing with 100 µL ice-cold acetone was performed. Cells were finally resuspended in 1.5 mL 1X PBS buffer and stained with the FM-1–43FX dye for 10 minutes in the dark. Cells were mounted on a coverslip coated with 10% poly-L-lysine and air-dried before observation under the microscope. The TIE-1 volume was determined as 1.048 *p*m^3^ using ~1,000 individual cells. PHA cell % vol/vol was obtained by dividing the volume of PHA per cell with the cell volume assuming that PHA density is 1.22 g/cm^3^ (71), and the cell % wt/wt fresh weight was obtained by dividing PHA mass by the fresh cell mass assuming that the cell density is 1.1 g/cm^3^ (72). The dry cell weight was estimated to be 40% of the fresh cell weight (73).

### Genomic DNA sequencing of the engineered RuBisCo strains *Ωrub(I)* and *Ωrub(I&II)*

DNA extracted from each strain was submitted to Plasmidsaurus. Bacterial genome sequencing was performed by Plasmidsaurus using Oxford Nanopore Technology. Raw reads were downloaded from Plasmidsaurus and were analyzed and annotated on KBase (74) using the following pipeline.

Read quality filtering was performed using Filtlong (75), keeping the best 90% of reads by quality score, with a minimum length of 1,000 bp. Quality control of filtered reads was performed using FastQC (76). Assembly of reads was performed using the long-read assemblers MiniASM (77) and Flye (78). Quality of assemblies was checked using Quast (79). Finished assemblies were annotated for features (e.g., genes and RNA-coding regions) using RAST (80). Genomes were uploaded to the RAST server (81) viewed using the SEED viewer (82), and construct integration sites were confirmed using BLAST (83).

## Data Availability

All data in the paper will be provided by authors upon request.

## SUPPLEMENTAL MATERIAL

Supplemental material aem.01438-24-s0001.docx Figures S1 to S5; Tables S1 to S5.

ASM does not own the copyrights to Supplemental Material that may be linked to, or accessed through, an article. The authors have granted ASM a non-exclusive, world-wide license to publish the Supplemental Material files. Please contact the corresponding author directly for reuse.
