# Supplementary material for: Overexpression of RuBisCO form I and II genes in Rhodopseudomonas palustris TIE-1 augments polyhydroxyalkanoate production heterotrophically and autotrophically
Source: Appl Environ Microbiol. 2024 Aug 20;90(9):e01438-24. doi: 10.1128/aem.01438-24 (PMC11409669; doi:10.1128/aem.01438-24)
Supplement: Supplemental material — Figures S1 to S5; Tables S1 to S5. [file aem.01438-24-s0001.docx]

**Supplementary Figures:**


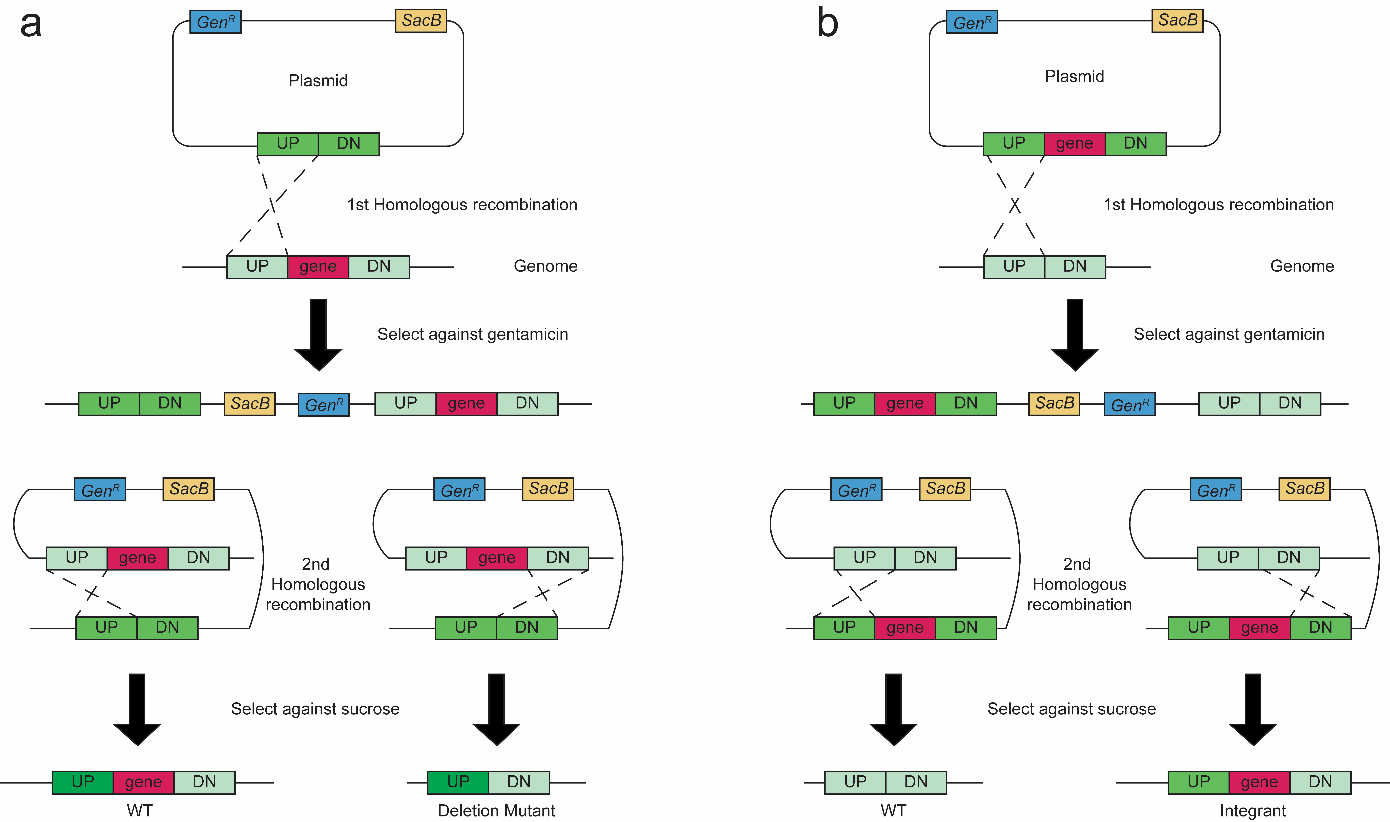


**Figure S1.**

**Figure S1.** Schematic of two-step integration. a. Schematic of generating a knockout mutant b. Schematic of generating a knock-in mutant UP: upstream homologous arm, DN: down-stream homologous arm, Gen^r^: gentamicin resistance, *SacB*: sucrose counter-selection marker


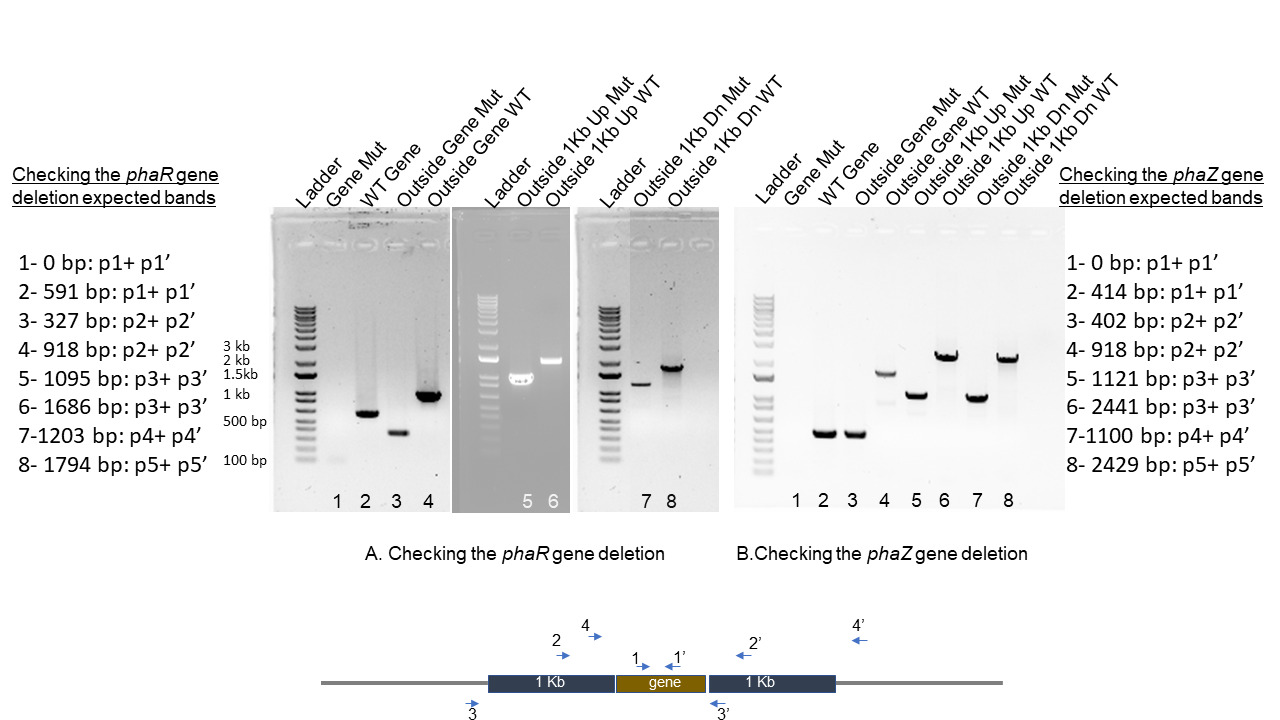
**Figure S2.**

**Figure S2.** Gel image displaying the anticipated sizes of amplicons from the primer sets (bottom figure) utilized to verify *ΔphaR* (left; A) and *ΔphaZ* (right; B). Mut = mutant, WT = wild type."

**Figure S3.**


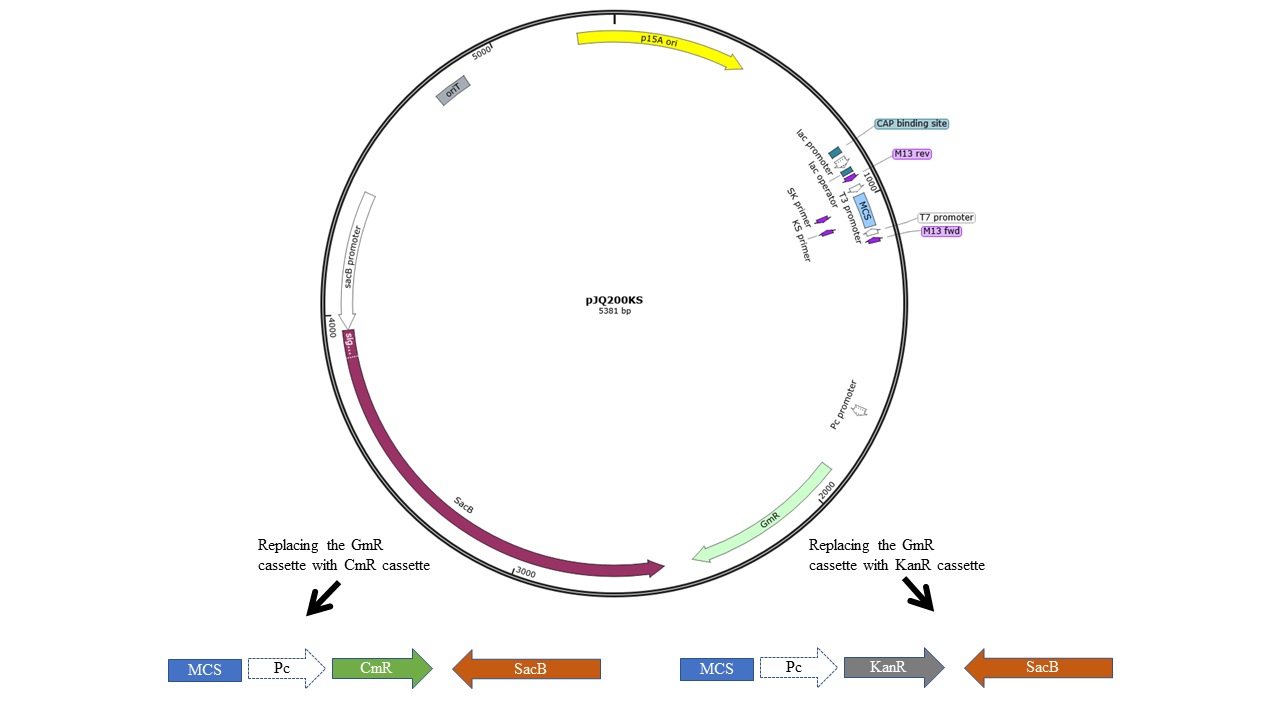


**Figure S3.** Creation of the two newly constructed pJQ200KS plasmids with gentamycin or kanamycin cassette **Gentamycin** (GmR) cassette of the pJQ200KS plasmid was replaced with either a kanamycin gene resistance cassette (KanR) or a chloramphenicol gene resistance cassette (CmR). MCS- Multiple Cloning Site. Pc-Pc- Pc promoter

**Figure S4.**

**
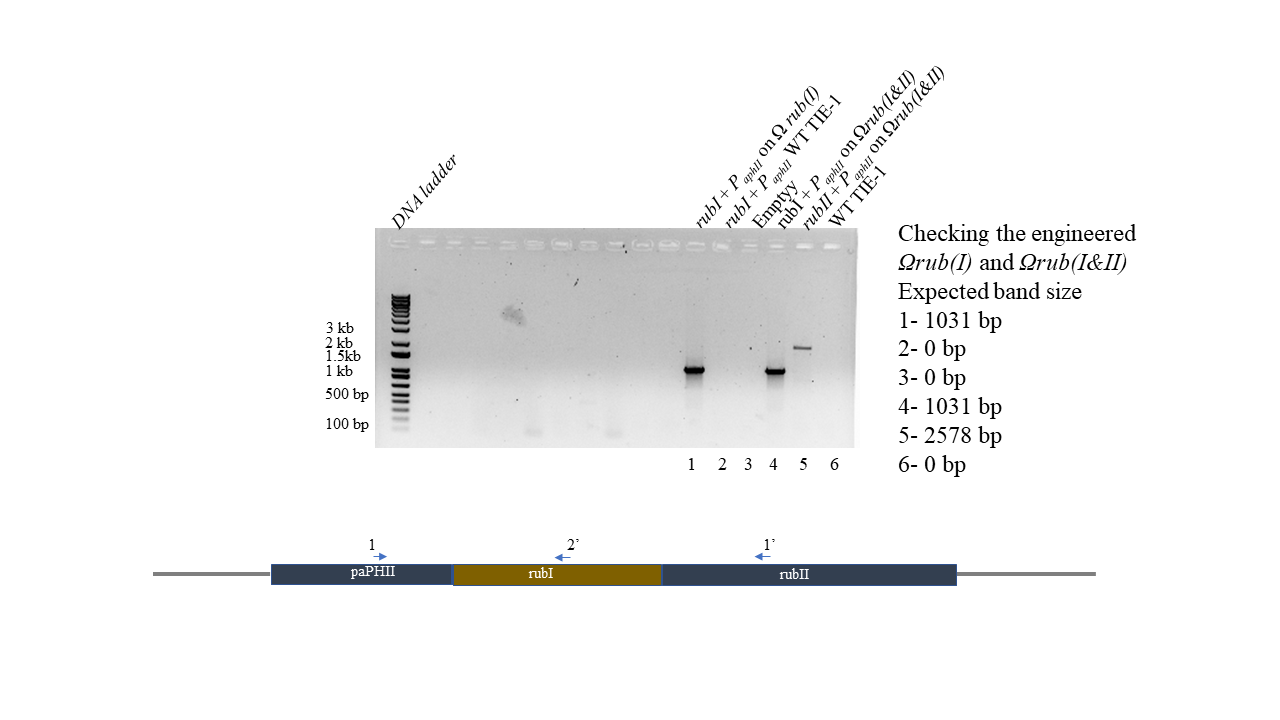
**

**Figure S4.** Gel image displaying the anticipated sizes of amplicons from the primer sets (bottom figure) utilized to verify the Ω*rub(I)* and Ω*rub(I&II)* engineered strains using PCR amplification spanning the promoter region *P_aphII_* to the integrated either Rubisco form I (*rubI*)or form II (*rubII*). The primers set used for the PCR amplifications are listed in Table S4.

**Figure S5.**

Genome 1 (“TIE-1 RuBisCO I and II”):

We expect to see each rubisco sequence TWICE — once in the native context, and then again under regulation of the *P_aphII_* promoter. We see *P_aphII_* followed by 3 RuBisCO genes, as expected.


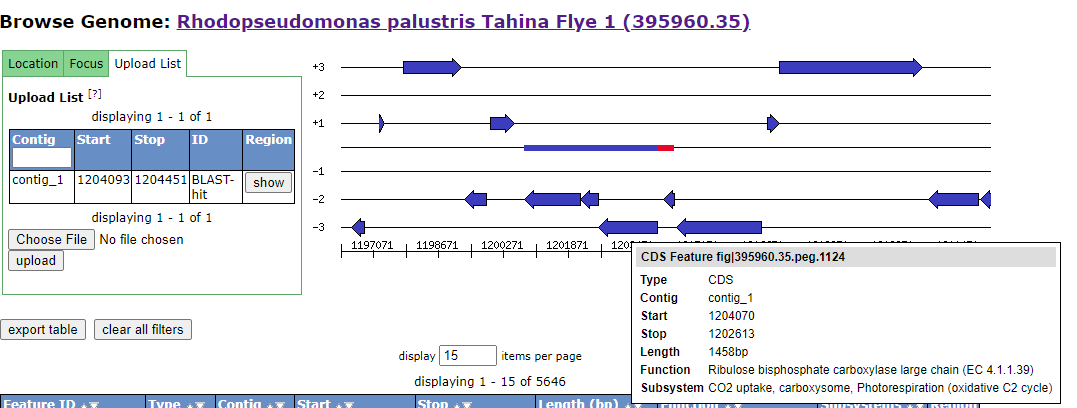


Construct integrated at location: 1204451 (start of *P_aphII_*)  – 1200785 (end of RuBisCO II)

Genome 2 (“TIE-1 RuBisCO I”):

As we expect, we see the RuBisCO sequence TWICE — once in the native context, and then again under regulation of the *P_aphII_* promoter. *P_aphII_* in red, then the two blue genes after it are the large and small chains of RuBisCO I.


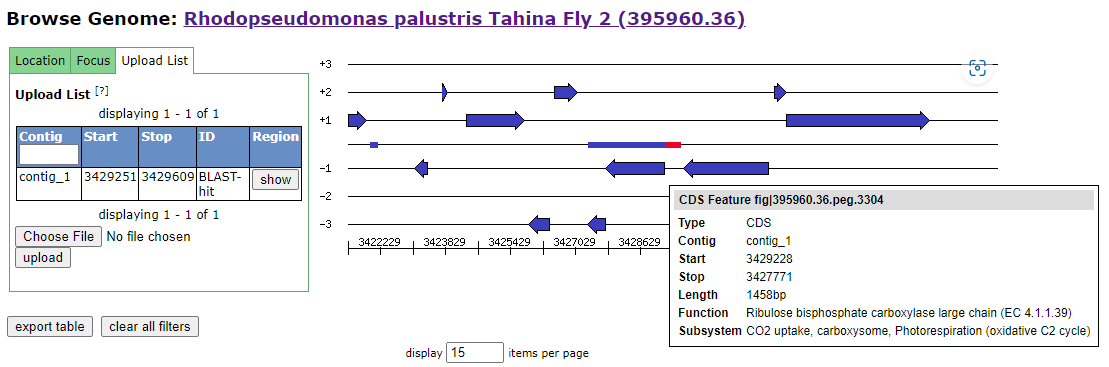


Construct integrated at location: 3429609 (start of *P_aphII_*) – 3427335 (end of RuBisCO I part 2)

Genome 3 (“TIE-1 *attB*”):

Full 34bp *attB* site ( GTGCCAGGGCGTGCCCTTGGGCTCCCCGGGCGCG ) present (red box in below image).  Full sequence did not appear in either of the two other genomes.  *P_aphII_* sequence did not appear in this genome. This genome also has a $\varphi$*C31* Integrase (blue arrow down and right from *attB*)


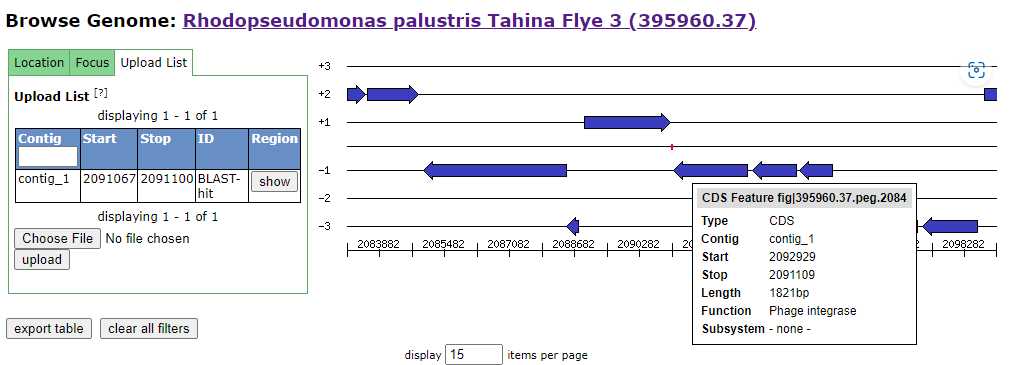


And a *lacI* gene (before the *φC31* integrase gene).  Between these two are the Lac promoter and the Lac operator, intact.


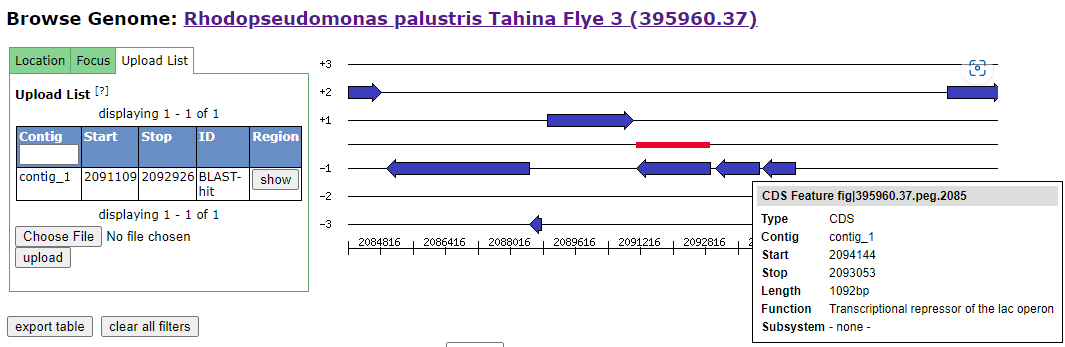


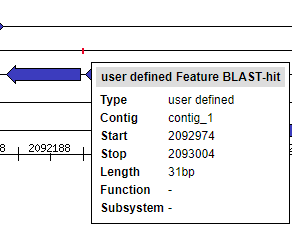

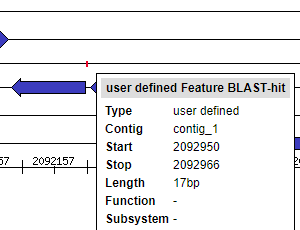


**Figure S5:** Genome sequencing figures showing the successful construction of the two TIE-1 strains Ω*rub(I)* and Ω*rub(I&II* ) carrying overexpression of the two RuBisCO form I and form II genes.

**Supplementary Tables:**

**Table S1. Growth parameters from 10mM butyrate**

|  | **Generation time g (h)** | | | | | | | | | | | | |
| --- | --- | --- | --- | --- | --- | --- | --- | --- | --- | --- | --- | --- | --- |
|  | *ΔphaR* | *p* | *ΔphaZ* | *p* | *Δgly* | *p* | *Δnif* | *p* | *Ωrub(I)* | *p* | *Ωrub(I&II)* | *p* | WT |
| But (NH_4_Cl) | 11.82(0.63) | 0.01 | 11.52(0.47) | 0.01 | 6.69(0.19) | 0.007 | 6.62(0.24) | 0.007 | 16.09(5.65) | 0.09 | 7.65(1.16) | 0.17 | 9.01(0.78) |
| But (N_2_) | 10.06(0.39) | 0.11 | 12.59(1.31) | 0.02 | 7.18(1.85) | 0.104 | NG | NG | 44.73(8.89) | 0.002 | 10.03(0.75) | 0.29 | 9.45(0.34) |
|  | lag time (h) | | | | | | | | | | | | |
| But (NH_4_Cl) | 72.17(3.48) | 0.0089 | 80.29(4.84) | 0.003 | 64.4(1.08) | 0.053 | 64.77(0.5) | 0.0395 | 110.91(23.6) | 0.01 | 62.86(0.49) | 0.11 | 58.73(3.44) |
| But (N_2_) | 86.11(1.27) | 0.0032 | 102.64(3.67) | 0.0005 | 80.3(4.22) | 0.972 | NG | NG | 284.8(37.04) | 0.001 | 76.25(1.1) | 0.01 | 80.39(0.91) |

But=butyrate, NG= No growth, WT= wild type TIE-1, standard deviations from biological triplicates are in (), *p* = *p* values

**Table S2. PHA productions obtained under different growth conditions.**

|  | PHA (mg/L/0D) | | | | | | | | | | | | |
| --- | --- | --- | --- | --- | --- | --- | --- | --- | --- | --- | --- | --- | --- |
| Growth conditions | *ΔphaR* | *p* | *ΔphaZ* | *p* | *Δgly* | *p* | *Δnif* | *p* | *Ωrub(I)* | *p* | *Ωrub(I&II)* | *p* | WT |
| Butyrate (NH_4_Cl) | 30.79(0.009) | 0.055 | 5.29(0.51) | 0.162 | 8.39(0.477) | 0.38 | 9.79(0.43) | 0.406 | 38.72(4.11) | 0.13 | 70.22(1.32) | 0.001 | 13.27(0.22) |
| Butyrate (N_2_) | 25.11(0.93) | 0.02 | 7.077(1.45) | 0.002 | 32.27(1.68) | 0.73 | NG | NG | 68.54(6.06) | 0 | 111.7(6.26) | 0.000 | 35.18(0.37) |
| H_2_ (NH_4_Cl) | 28.81(1.91) | 0.098 | 31.49(2.47) | 0.057 | 42.81(0.16) | 0.056 | 46.08(3.61) | 0 | 59.51(2.84) | 0 | 40.02(3.61) | 0.012 | 20.23(0.59) |
| H_2_ (N_2_) | 64.88(3.03) | 0.517 | 51.36(2.14) | 0.935 | 22.09(0.98) | 0.098 | NG | NG | 54.1(32.11) | 0.862 | 23.12(4.22) | 0.111 | 49.96(0.58) |
| Fe(II) (NH_4_Cl) | 30.79(0.85) | 0 | ND | N/A | 25.21(0.77) | 0 | 58.04(0.42) | 0.37 | 44.62(0.94) | 0.033 | 74.93(3.53) | 0.98 | 74.65(1.23) |
| Fe(II) (N_2_) | 11.37(0.49) | 0.082 | 6.03(0.63) | 0.004 | 11.89(0.22) | 0.075 | NG | NG | 5.26(0.50) | 0.002 | 4.96(0.12) | 0.01 | 22.0(1.57) |
| PE (NH_4_Cl) | 1.42(0.102) | 0.005 | 7.73(0.25) | 0.80 | 4.45(1.21) | 0.08 | 3.42(0.03) | 0.01 | 2.48(0.54) | 0.018 | 2.35(0.082) | 0.005 | 7.42(0.08) |
| PE (N_2_) | 1.64(0.12) | 0.89 | 4.59(0.311) | 0.003 | 6.54(0.48) | 0.011 | NG | NG | 4.08(1.51) | 0.05 | 2.75(0.09) | 0.05 | 1.54(0.21) |
|  | PHA (mg/L/cell/h)(10^-13^) | | | | | | | | | | | | |
| Butyrate (NH_4_Cl) | 2.23(0) | 0.198 | 0.67(0.06) | 0.163 | 0.65(0.056) | 0.155 | 1.23(0.04) | 0.327 | 3.64(0.19) | 0.201 | 8.27(0.1) | 0.05 | 2.239(0.04) |
| Butyrate (N_2_) | 2.42(0.09) | 0.025 | 1.85(0.23) | 0.286 | 1.96(0.11) | 0.073 | NG | NG | 3.44(0.21) | 0.555 | 11.69(0.46) | 0.00 | 3.479(0.04) |
| H_2_ (NH_4_Cl) | 2.97(0.19) | 0.073 | 3.6(0.25) | 0.108 | 4.19(0.016) | 0.056 | 4.75(0.37) | 0 | 4.65(0.27) | 0.05 | 3.31(0.29) | 0.026 | 1.98(0.05) |
| H_2_ (N_2_) | 1.27(0.05) | 0.576 | 0.2(0.08) | 0.022 | 0.43(0.019) | 0.057 | NG | NG | 1.24(0.024) | 0.704 | 0.69(0.07) | 0.279 | 1.032(0.01) |
| Fe(II) (NH_4_Cl) | 1.53(0.04) | 0.00E+00 | ND | N/A | 2.01(0.061) | 0 | 7.01(0.05) | 0.644 | 4.83(0.1) | 0.31 | 6.033(0.28) | 0.98 | 6.011(0.09) |
| Fe(II) (N_2_) | 0.5(0.02) | 0.025 | 0.26(0.02) | 0.01 | 0.74(0.016) | 0.088 | NG | NG | 0.59(0.05) | 0.053 | 0.68(0) | 0.088 | 1.769(0.08) |
| PE (NH_4_Cl) | 0.031(0.006) | 0.006 | 0.17(0.005) | 0.8 | 0.099(0.02) | 0.08 | 0.076(0.00) | 0.01 | 0.055(0.014) | 0.01 | 0.05(0.001) | 0.005 | 0.16(0.001) |
| PE (N_2_) | 0.036(0.002) | 0.89 | 0.103(0.00) | 0.023 | 0.14(0.01) | 0.011 | NG | NG | 0.09(0.03) | 0.05 | 0.06(0.002) | 0.05 | 0.034(0.004) |
| PHA (Cell % v/v) | | | | | | | | | | | | | |
| Butyrate (NH_4_Cl) | 1.81(0) | 0 | 0.061(0.02) | 0.051 | 0.08(0.08) | 0.056 | 0.078(0.02) | 0.054 | 2.27(0.17) | 0.007 | 4.13(0.078) | 0.001 | 0.78(0.007) |
| Butyrate (N_2_) | 1.47(0.05) | 0.02 | 1.47(0.17) | 0.606 | 1.09(0.09) | 0.374 | NG | NG | 4.03(O.35) | 0 | 6.57(0.36) | 0 | 2.07(0.015) |
| H_2_ (NH_4_Cl) | 1.69(0.10) | 0.098 | 2.05(0.14) | 0.127 | 2.51(0.007) | 0.056 | 2.71(0.21) | 0 | 2.8(0.16) | 0.055 | 2.35(0.21) | 0.012 | 1.18(0.03) |
| H_2_ (N_2_) | 3.82(0.17) | 0.517 | 3.0(0.11) | 0.952 | 1.29(0.05) | 0.098 | NG | NG | 3.18(0.14) | 0.862 | 1.36(0.242) | 0.111 | 2.94(0.03) |
| Fe(II) (NH_4_Cl) | 1.81(0.05) | 0 | ND | N/A | 1.48(0.045) | 0 | 3.42(0.024) | 0.375 | 2.63(0.05) | 0.033 | 4.41(0.20) | 0.98 | 4.40(0.072) |
| Fe(II) (N_2_) | 0.67(0.028) | 0.082 | 0.35(0.036) | 0.004 | 0.70(0.023) | 0.075 | NG | NG | 0.27(0.002) | 0.002 | 0.53(0.007) | 0.111 | 1.30(0.068) |
| PE (NH_4_Cl) | 0.078(0.01) | 0.006 | 0.3910649 | 0.803 | 0.27(0.07) | 0.085 | 0.19(0.001) | 0.25 | 0.14(0.03) | 0.01 | 0.13(0.004) | 0.005 | 0.43(0.005) |
| PE (N_2_) | 0.09(0.007) | 0.89 | 0.26(0.017) | 0.023 | 0.38(0.02) | 0.011 | NG | NG | 0.24(0.08) | 0.05 | 0.16(0.005) | 0.05 | 0.08(0.01) |
| Cell % PHA dry weight/fresh cell weight) | | | | | | | | | | | | | |
| Butyrate (NH_4_Cl) | 2.01(0) | 0 | 0.06(0.02) | 0.051 | 0.095(0.09) | 0.056 | 0.086(0.02) | 0.054 | 2.52(0.19) | 0.007 | 4.58(0.08) | 0.001 | 0.86(0.008) |
| Butyrate (N_2_) | 1.63(0.06) | 0.02 | 1.63(0.1) | 0.606 | 1.21(0.104) | 0.374 | ND | NG | 4.47(0.39) | 0 | 7.29(0.4) | 0 | 2.29(0.01) |
| H_2_ (NH_4_Cl) | 1.88(0.121) | 0.098 | 2.28(0.15) | 0.127 | 2.79(0.0086) | 0.056 | 3.01(0.23) | 0 | 3.1(0.18) | 0.055 | 2.61(0.23) | 0.012 | 1.31(0.03) |
| H_2_ (N_2_) | 4.24(0.19) | 0.517 | 3.33(0.13) | 0.952 | 1.43(0.06) | 0.098 | ND | NG | 3.53(0.15) | 0.862 | 1.50(0.2) | 0.111 | 3.26(0.04) |
| Fe(II) (NH_4_Cl) | 2.01(0.055) | 0 | ND | N/A | 1.64(0.05) | 0 | 3.79(0.027) | 0.375 | 2.91(0.06) | 0.033 | 4.8(0.23) | 0.98 | 4.88(0.080) |
| Fe(II) (N_2_) | 0.74(0.032) | 0.082 | 0.39(0.040 | 0.004 | 0.77(0.026) | 0.075 | ND | NG | 0.308(0.032) | 0.002 | 0.59(0.007) | 0.111 | 1.44(0.075) |
| PE (NH_4_Cl) | 0.086(0.01) | 0.006 | 0.43(0.01) | 0.803 | 0.303(0.07) | 0.085 | 0.21(0.001) | 0.25 | 0.15(0.03) | 0.01 | 0.14(0.005) | 0.005 | 0.47(0.005) |
| PE (N_2_) | 0.10(0.00) | 0.89 | 0.29(0.01) | 0.023 | 0.42(0.03) | 0.011 | ND | NG | 0.26(0.095) | 0.05 | 0.17(0.006) | 0.05 | 0.095(0.013) |

Values are averages from biological triplicate except for those from photoelectrotrophic (PE) growth conditions which are duplicates. Standard error values are in (). *p* = *p* values against wild type values, Fe= Iron, NH_4_Cl= Ammonium chloride, N_2_=Nitrogen gas, PE= photoelectrotrophic growth, NG= No growth, ND= Not detectable

**Table S3.** List of antibiotics and sucrose used in this study and their respective concentration.

| Antibiotic | Concentration (μg/mL) for *E. coli* | Concentration (μg/mL) for TIE-1 |
| --- | --- | --- |
| Gentamycin | 20 | 200 |
| Chloramphenicol | 25 | 100 |
| Ampicillin | 100 | NA |
| Kanamycin | 50 | 50 |
| Tetracycline | 10 | NA |
| Sucrose | 100000 | 100000 |

**Table S4.** List of primers used to design plasmids used in this study

**Table S5.** List of primers used to design and check the mutants constructed in this study.

| Primer names | Sequence |
| --- | --- |
| Primers used to design and to check the *phaR* mutant | |
| *phaR* 1kb DN BamHI Fw 3 | tagttaGGATCCCAGCGAGGTTTGCTGCTTAG |
| *phaR* 1kb DN PstI Rev 4 | tcatcgCTGCAGATCGGGCCCGGTATCGCTCAC |
| *phaR* 1kb up BamHI Rev 2 | tcagtaGGATCCGTCACCCACACACACGGAGGA |
| *phaR* 1kb up SpeI Fw 1 | tacgtaACTAGTTCTTGCCGAGCTTCTCCGCCTGCTT |
| *phaR* gene Check Fw | ATGGCGAAATCAGACCAACCGAC |
| *phaR* gene Check Rv | CTATTCGTCCTTCTTCGGCTGT |
| *phaR* Check Fw | GTCAATCCGGCACACGCATCT |
| *phaR* Check Rev | GCAAAGCCGTCAATTAGCAAG |
| *phaR* 1kb Up check Fw | TCGTTGATTCCCGACGCAGAAC |
| *phaR* 1kb Up Check Rev | TCGAAGACATCCGGTTCTGACATG |
| *phaR* 1kb Dn Check Fw | CTTATCCTCCGTGTGTGTG |
| *phaR* 1kb Dn check Rev | CGGACTGGTGCTGAAGAACT |
| Primers used to design and to check the *phaZ* mutant | |
| *phaZ* 1Kb DN BamHI Rev 3 | ctacgtGGATCCTTACTACGCGTCGTCGTTAGCTG |
| *phaZ* 1Kb DN SpeI Rev 4 | tgagtACTAGTAGGTCGTTGCCGTAGGAGTCGA |
| *phaZ* 1Kb Up BamHI Rev 2 | tactctGGATCCCGTGTCGTGTCCCTCGTAAC |
| *phaZ* 1Kb Up SpeI Fw 1 | gtcatgACTAGTAGGTCGCATCTGGAAAGTTATCGC |
| *phaZ* gene Check Fw | CCCAACTGCTTCAACCTG |
| *phaZ* gene Check Rv | ATAGCCGCAGACCTCGTTCT |
| *phaZ* Check Fw | CGAATTCGTCCACGATGAG |
| *phaZ* Check Rev | TCGTCGACACCGAGAATG |
| *phaZ* check 1Kb Dn Fw | TGTGGACTGTCCGTGCTTAC |
| *phaZ* check 1Kb Dn Rev | AGTGGTTCATCTCGACCAGATG |
| *phaZ* check 1Kb Up Rev | ATGTACACAAGTCATCAGGATG |
| *phaZ* check 1Kb up Fw | AGGAGGTGTTTCTCGACGTC |

**Table S5.** Primers used to check the insertion of RuBisCO I and II in TIE-1 used in this study.

| Primer name | Sequence |
| --- | --- |
| *P_aphII_* Check Fw (1) | GTAGAAAGCCAGTCCGCAGAA |
| RuBisCO I Check Rev (2) | GGCGGGACATAGAAGTCGTA |
| RuBisCO II Check Rev (1’) | TGTAGCCGATCACGAGGTC |

**φC31 integrase strain construction**

The φC31 *attB* and *attP* sequences are obtained from the previously published sequences [59]. The *attP* sequence was cloned into pJQ200KS, resulting in pWB081. Then the *P_aphII_*-*mCherry*-fd cassette was cloned into pJQ200KS, resulting in pWB081. For the plasmid-based system, the φC31 integrase sequence was cloned into either plasmid pSRKGm or pWB081, resulting in pWB084 and pWB088. For the genome-based system, the φC31 integrase sequence was cloned to pAB314, resulting in pWB089. The *attB* sequence was also cloned into pAB314, resulting in pWB083.

*Integration system in TIE-1:* We explored four φC31 expression systems: inducible plasmid-based, constitutive genome-based (although not viable), and constitutive plasmid-based for φC31 integrase expression to determine the optimal integration system. We observed that the inducible genome-based system proved to be most efficient. However, incorporating φC31 integrase into genomes of organisms poses significant challenges. Thus, using plasmid-based systems are significantly more straightforward. Between the two plasmid-based systems, using a constitutive promoter to drive φC31integrase expression resulted in higher efficiency and did not leave the suicide plasmid behind. Thus, the plasmid-based system with the constitutive promoter stands out as an easy-to-use and efficient approach for integration using φC31 integrase in TIE-1.
